# Defining early steps in *Bacillus subtilis* biofilm biosynthesis

**DOI:** 10.1128/mbio.00948-23

**Published:** 2023-08-31

**Authors:** Christine A. Arbour, Rupa Nagar, Hannah M. Bernstein, Soumi Ghosh, Yusra Al-Sammarraie, Helge C. Dorfmueller, Michael A. J. Ferguson, Nicola R. Stanley-Wall, Barbara Imperiali

**Affiliations:** 1 Department of Biology and Department of Chemistry, Massachusetts Institute of Technology, Cambridge, Massachusetts, USA; 2 Division of Molecular Microbiology, School of Life Sciences, University of Dundee, Dundee, United Kingdom; 3 Wellcome Centre for Anti-Infectives Research, School of Life Sciences, University of Dundee, Dundee, United Kingdom; University of Washington, Seattle, Washington, USA; Universiteit Leiden, Leiden, Netherlands

**Keywords:** chemoenzymatic synthesis, bacillosamine, genetic complementation, biofilm

## Abstract

The *Bacillus subtilis* extracellular biofilm matrix includes an exopolysaccharide (EPS) that is critical for the architecture and function of the community. To date, our understanding of the biosynthetic machinery and the molecular composition of the EPS of *B. subtilis* remains unclear and incomplete. This report presents synergistic biochemical and genetic studies built from a foundation of comparative sequence analyses targeted at elucidating the activities of the first two membrane-committed steps in the EPS biosynthetic pathway. By taking this approach, we determined the nucleotide sugar donor and lipid-linked acceptor substrates for the first two enzymes in the *B. subtilis* biofilm EPS biosynthetic pathway. EpsL catalyzes the first phosphoglycosyl transferase step using uridine diphosphate (UDP)-di-*N*-acetyl bacillosamine as phospho-sugar donor. EpsD is a predicted GT-B fold (GT4 family) retaining glycosyl transferase that catalyzes the second step in the pathway that utilizes the product of EpsL as an acceptor substrate and UDP-*N*-acetyl glucosamine as the sugar donor. Thus, the study defines the first two monosaccharides at the reducing end of the growing EPS unit. In doing so, we provide the first evidence of the presence of bacillosamine in an EPS synthesized by a Gram-positive bacterium.

**IMPORTANCE:**

Biofilms are the communal way of life that microbes adopt to increase survival. Key to our ability to systematically promote or ablate biofilm formation is a detailed understanding of the biofilm matrix macromolecules. Here, we identify the first two essential steps in the *Bacillus subtilis* biofilm matrix exopolysaccharide (EPS) synthesis pathway. Together, our studies and approaches provide the foundation for the sequential characterization of the steps in EPS biosynthesis, using prior steps to enable chemoenzymatic synthesis of the undecaprenyl diphosphate-linked glycan substrates.

## INTRODUCTION

Biofilms are self-associating microbial systems that contain surface-adherent individuals within an extracellular matrix (1). The nonpathogenic bacterium, *Bacillus subtilis* (*Bs*), has been used extensively for understanding biofilm formation due to its ease of genetic manipulation and its extensive applied uses across diverse sectors of our economy (2). The *B. subtilis* biofilm matrix contains multiple specific components: BslA (a hydrophobin-like protein that confers hydrophobicity and structure to the community), fibers of the protein TasA (required for the structural integrity of biofilm), extracellular DNA (eDNA, important at early stages of biofilm formation), poly-γ-glutamic acid (possible function in water retention), and an exopolysaccharide (EPS) (3).

**Fig 1:**
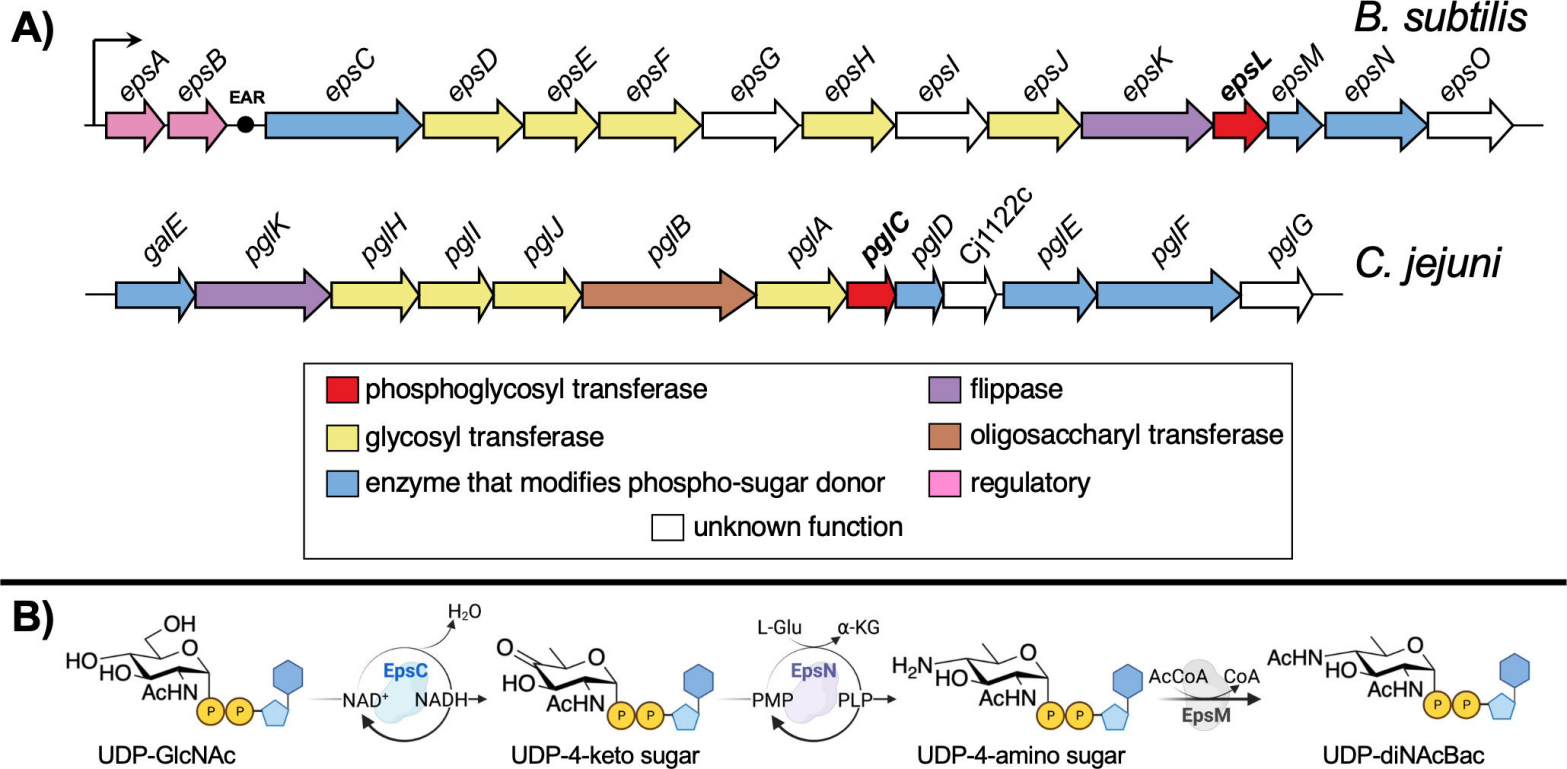
Comparison of glycoconjugate synthesis in *B. subtilis* and *C. jejuni*. (**A**) The *epsA-O* operon of *B. subtilis* and the *pgl* operon of *C. jejuni* drawn broadly to scale. EAR represents the *eps*-associated RNA (6) situated between *epsB* and *epsC*. (**B**) The biosynthesis of UDP-diNAcBac in *B. subtilis* catalyzed by EpsCNM. EpsC catalyzes the NAD^+^-dependent elimination of water across C5 and C6, while oxidizing C4 of UDP-GlcNAc. EpsN is a pyridoxal 5′-phosphate (PLP)-dependent aminotransferase. EpsM is an acetyltransferase that transfers an acetyl group from acetyl coenzyme A (AcCoA) onto UDP-4-amino sugar to provide UDP-diNAcBac (7 – 9).

The EPS is the main carbohydrate component of the *B. subtilis* matrix and is critical for biofilm architecture and biofilm function (4, 5). Despite considerable interest in understanding biofilm biosynthesis and regulation, the individual building blocks for this macromolecular glycoconjugate have not been determined. Biosynthesis of EPS is dependent on enzymes expressed from a 15-gene *epsABCDEFGHIJKLMNO* (*epsA-O*) operon (10), which has a similarity with the *Campylobacter jejuni pgl* operon (Fig. 1A). These enzymes have been annotated based on sequence analysis as a phosphoglycosyl transferase (PGT), glycosyl transferases (GTs), uridine diphosphate sugar (UDP-sugar) modifiers, a regulatory enzyme, and a flippase (5, 11, 12). However, most of the membrane-associated enzymes that are involved in the biosynthesis of exopolysaccharide in *B. subtilis* have not been biochemically characterized. Furthermore, analysis of EPS composition has afforded conflicting information. Even studies of the same strain of *B. subtilis* (namely, NCIB 3610) provided different carbohydrate compositions depending on the bacterial growth conditions and/or methods of extraction and purification. For example, when grown in glutamic acid and glycerol-rich media, an EPS fraction contained glucose, *N*-acetylgalactosamine (GalNAc), and galactose (Gal) (13, 14). The same strain grown in lysogeny broth (LB) media that included magnesium and manganese divalent cations produced an EPS fraction containing mannose and glucose (15, 16). Furthermore, growth in a minimal media supplemented with glucose (MMG) produced an EPS fraction containing poly-*N*-acetylglucosamine (GlcNAc) (5).

UDP-*N,N*′-diacetylbacillosamine (UDP-diNAcBac) is a prokaryote-specific nucleotide sugar donor (17). The monosaccharide component, diNAcBac, was originally discovered in *B. licheniformis* (18). Based on *in vitro* activity and sequence similarity, EpsC, EpsN, and EpsM are proposed to produce UDP-diNAcBac in *B. subtilis* (Fig. 1B) (7 – 9). To support the assignment of these Eps enzymes, isofunctional homologs in *Campylobacter*, in particular *C. jejuni* (PglF, PglE, and PglD) (Fig. 1A), have been biochemically characterized and shown to make UDP-diNAcBac in a similar fashion (17, 19 – 21). EpsCNM from *B. subtilis* and PglFED from *C. jejuni* (*Cj*) have 54%, 64%, and 50% sequence similarity, respectively (8).

Our overarching goal is to elucidate the composition and structure of the *B. subtilis* biofilm matrix EPS. Given the inconsistencies obtained from direct analysis of the extracted EPS material, we elected to start by determining the identity of the individual monosaccharides at the reducing end of the EPS. In this work, we investigate and define the substrate specificity of two enzymes encoded within the *eps* operon, EpsL and EpsD, annotated as a PGT and GT, respectively, using biochemical and genetic complementation approaches. We present experimental evidence supporting the designation of EpsL as a PGT, which installs diNAcBac as the first monosaccharide onto a undecaprenyl phosphate (UndP) carrier. We also identify EpsD as the second enzyme, and the first GT, in the pathway that likely installs GlcNAc onto the diNAcBac-appended lipid anchor. Thus, a key polyprenol-diphosphate-linked disaccharide is proposed and can be made available through chemoenzymatic synthesis. Therefore, our work sets the stage for future analysis of downstream glycosyltransferase reactions in the EPS pathway.

## RESULTS

### Characterizing the PGT (EpsL) in the EPS biosynthetic pathway

**Fig 2:**
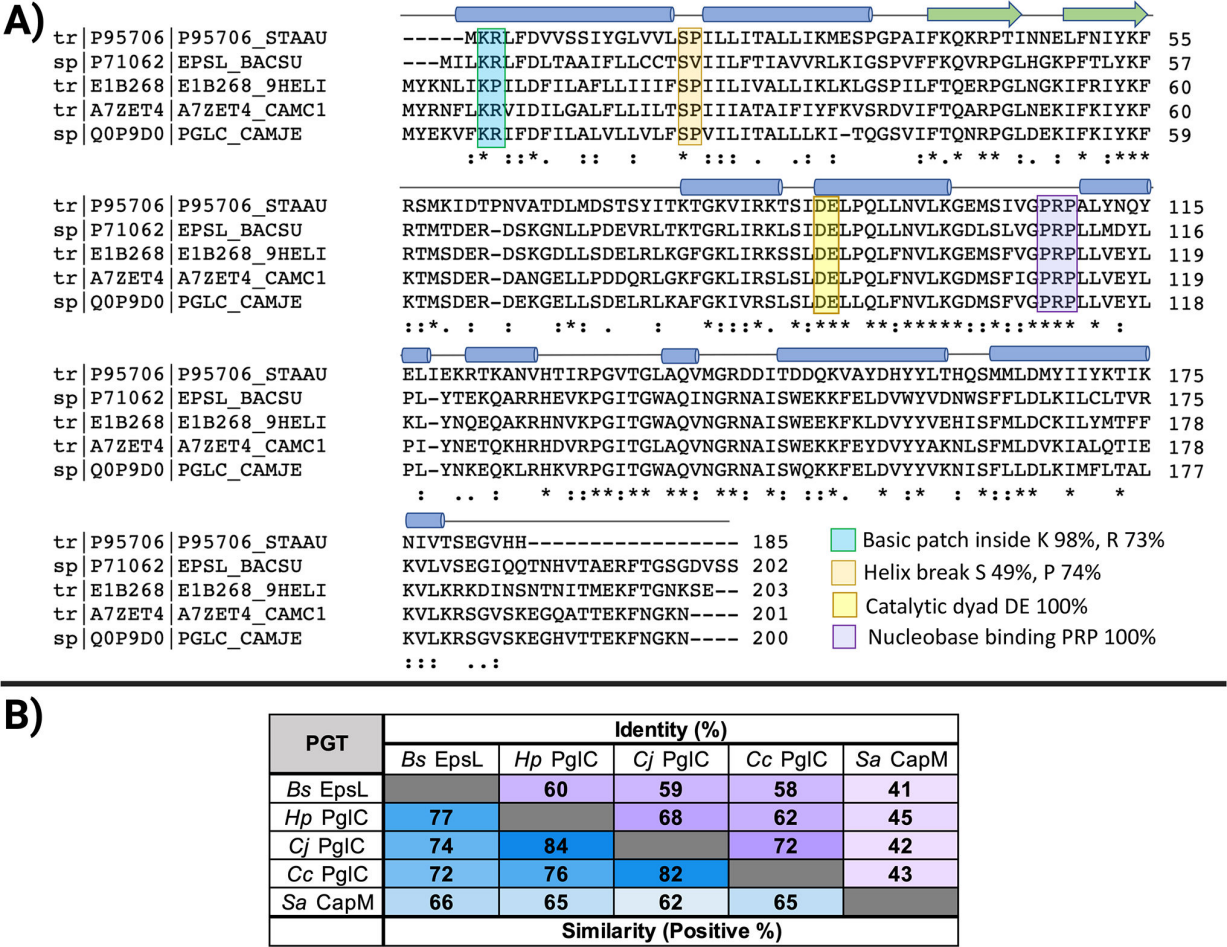
Protein sequence comparison of select mono-PGTs. (**A**) Sequence alignment of *Bs* EpsL with mono-PGTs from Gram-positive and Gram-negative bacteria. The percent conservation of key residues of interest is taken from reference (25) and is based on the alignment of 15,000 nonredundant sequences (27). (**B**) The basic local alignment search tool was used to obtain percent identity and similarity from accession numbers: *Bs* EpsL (P71062), *Hp* PglC (E1B268), *Cj* PglC (Q0P9D0), *Cc* PglC (A7ZET4), and *Sa* CapM (P95706).

PGTs are enzymes responsible for catalyzing the first membrane-committed step in many essential glycosylation pathways by transferring a sugar phosphate onto a lipid acceptor carrier. PGTs are represented by two distinct membrane topologies, mono- and polytopic (22), and perform mechanistically distinct modes of catalysis (23). The monotopic phosphoglycosyl transferases (mono-PGTs) comprise three families: small, long, and bifunctional enzymes. The sequence similarity network of small mono-PGTs provided an uncharacterized enzyme from *B. subtilis*, EpsL (24). *B. subtilis* EpsL contains the key residues that are the hallmarks of the mono-PGTs catalytic domain and other signature motifs (Fig. 2A) (25). These include a basic motif near the N-terminus and helix-break-helix motif in the membrane-associated domain that contribute to the membrane reentrant topology of the enzyme. Additionally, the catalytic dyad (DE) that is responsible for covalent catalysis and the uridine-binding residues (PRP) are present. Furthermore, EpsL is similar to small mono-PGTs from other Gram-positive bacteria (*Staphylococcus aureus* (*Sa*) 41% identity), a PGT that has been shown to use UDP-D-FucNAc as the sugar-phosphate donor substrate (26). However, higher-sequence similarity is observed with PglCs from *Campylobacter* [*C. concisus* (*Cc*) 58%, *C. jejuni* 59% identity] and *Helicobacter pullorum* (*Hp*) (60% identity) (Fig. 2B). Based on sequence similarity with mono-PGTs from *C. concisus* and *C. jejuni*, we hypothesized that EpsL uses UDP-diNAcBac. This is consistent with the conclusion that EpsCNM synthesize this particular UDP-sugar (7 – 9).

### Biochemical and genetic evaluation of EpsL substrate specificity

**Fig 3:**
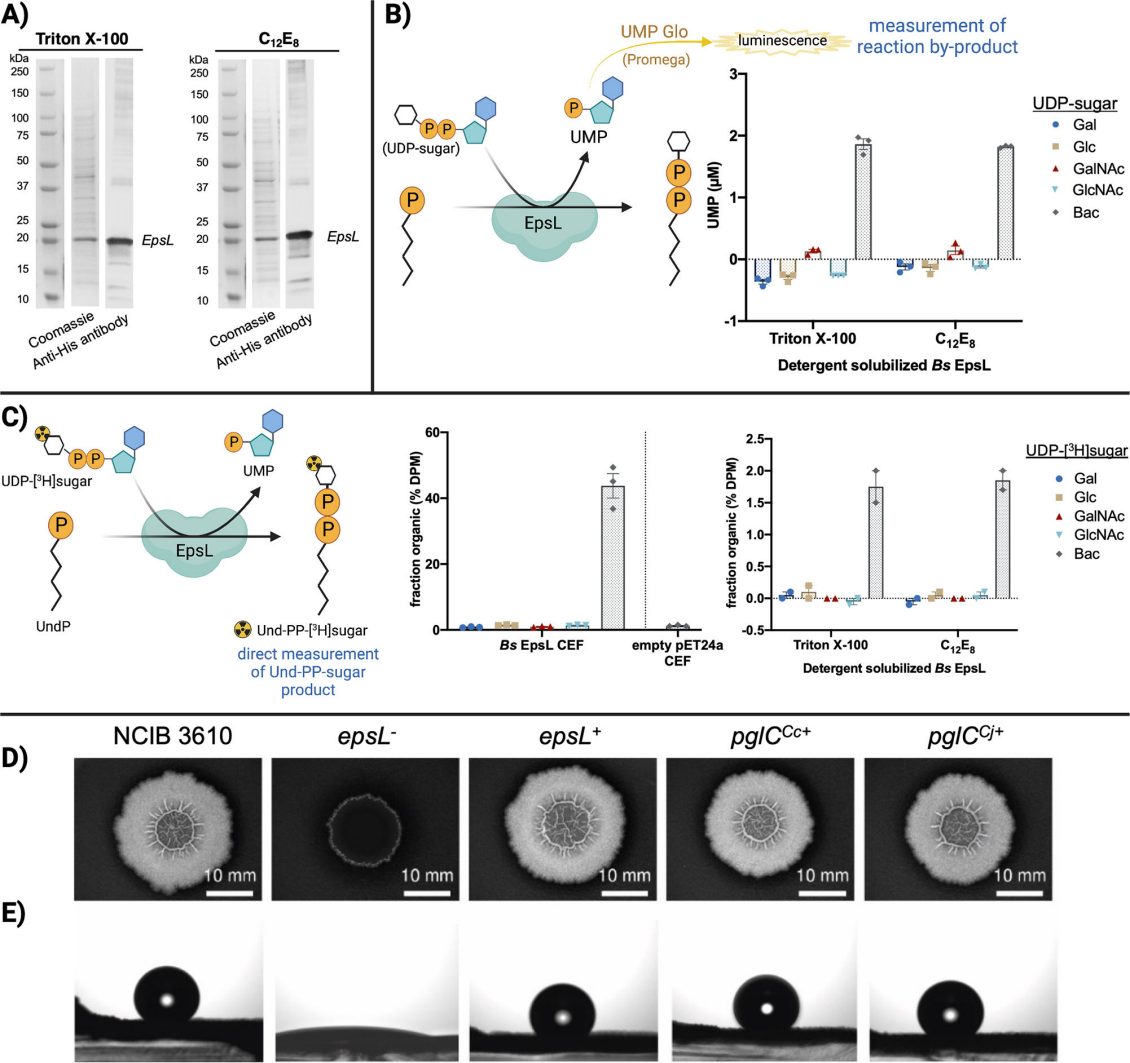
Purification and biochemical and phenotypic characterization of EpsL. (**A**) *B. subtilis* EpsL purification visualized by SDS-PAGE (Coomassie) and anti-His antibody Western blots. The same Precision Plus Proteins Standards lane was used for both panels. (**B**) Complementary biochemical activity assays of *B. subtilis* EpsL using UMP Glo, a luminescence-based assay that measures the UMP by-product of the PGT reaction. Error bars are given for mean ± SEM, *n* = 3. (**C**) A radioactivity-based assay that measures the Und-PP-[^3^H]sugar product. EpsL (*Bs*) activity is background subtracted and reported as the percentage of disintegrations per minute in the organic layer normalized to the total disintegrations per minute per quenched point. A negative control of CEF prepared from cells carrying an empty pET24a vector was assayed in parallel. Error bars are given for mean ± SEM (for CEF experiments, *n* = 3, and for detergent-solubilized *Bs* EpsL, *n* = 2). (**D**) and (**E**) Genetic complementation of *ΔepsL-Bs* mutant with *pglC* of *Campylobacter*. (**D**) represents colony biofilm morphologies of wild-type (*B. subtilis* NCIB 3610) *ΔepsL* mutant (*epsL* ^−^—NRS5907) and genetically complemented strains (*epsL* ^+^—NRS5942, *pglC^Cc^* ^+^—NRS6692, *pglC^Cj^* ^+^—NRS6618, (see Table S1). The colony biofilms were grown at 30°C for 48 h prior to imaging. (**E**) represents the respective sessile water drop analysis of the colony biofilms with a 5 µL water droplet on top. The representative images were taken after 5 min, except *epsL* ^−^ where the image was taken at 0 min due to extreme hydrophilicity of the surface in the absence of biofilm.

To test the hypothesis that EpsL uses UDP-diNAcBac as the phospho-sugar donor substrate, heterologous expression of *epsL* was carried out in *Escherichia coli* following a previously described protocol for monotopic PGTs from *C. concisus* and *C. jejuni* (23, 27, 28). After isolation of the cell envelope fraction (CEF), eight detergents were screened to evaluate the solubilization efficiency and purity of the enzyme (Fig. S1A and B). The detergent solubilization screen provided two detergents, Triton X-100 and octaethylene glycol monododecyl ether (C_12_E_8_), that efficiently solubilized EpsL while minimizing the solubilization of undesired proteins from the cell envelope fraction. For that reason, EpsL was solubilized and purified in Triton X-100 and C_12_E_8_ on a preparative scale for downstream applications (Fig. 3A; Fig. S1C).

The activity of solubilized and purified EpsL was evaluated. This was achieved through a substrate screen with five UDP-sugar donors and UndP as a lipid acceptor using two complementary biochemical assays; UMP Glo and a radioactivity-based assay (Fig. 3B and C). The standard commercial ^3^H-labeled and unlabeled UDP-sugars (UDP-Gal, UDP-Glc, UDP-GalNAc, and UDP-GlcNAc) were used for the screens. Additionally, UDP-diNAcBac and UDP-[^3^H]diNAcBac, both prepared via chemoenzymatic methods, were used (Fig. S2A). The UMP Glo assay developed by Promega monitors the production of UMP over the course of a reaction (Fig. 3B) (29). This indirect measurement of reaction progress is excellent for initial screens of PGTs. However, to quantify the reaction more specifically, an assay that monitors the main reaction product was needed. Therefore, we employed a radioactivity-based assay to directly measure the formation of the Und-PP-sugar following liquid-liquid extraction of the Und-PP-linked product. This radioactivity-based assay was performed on both the CEF containing *Bs* EpsL and detergent-solubilized and partially purified enzyme (Fig. 3C; Fig. S1C). We observed a clear preference for UDP-diNAcBac as substrate using both methods. In addition, to establish the presence or absence of off-target effects, we performed assays on CEF prepared from cells that carried the empty pET24a vector (Fig. S1D; Fig. 3C). In comparison to the activity of the CEF with the solubilized and partially purified enzyme, we note that EspL loses considerable activity on solubilization. This is not uncommon with membrane proteins, in general, and has been observed with most of the PGT studied so far (30). We additionally monitored reaction progress in nonradioactive reactions by normal phase silica thin-layer chromatography (TLC) (Fig. S2B). During the reaction, a new product was formed that had the same retention factor (*R*_*f*_) as the authentic standard Und-PP-diNAcBac from *C. concisus* PglC (31), providing biochemical evidence that EpsL can use UDP-diNAcBac as donor substrate in the presence of the UndP acceptor. Moreover, defined sequence fingerprint regions are associated with mono-PGTs that show UDP-diNAcBac substrate specificity, the assignment of the UDP-diNAcBac as the substrate of EpsL is consistent with these sequence motifs (32).

We proposed that if EpsL was a PGT that installs diNAcBac as the first monosaccharide in the EPS pathway, then, PglC of *Campylobacter* should be able to substitute for EpsL activity *in vivo*. In the absence of *epsL*, *B. subtilis* is unable to form the rugose, hydrophobic colony biofilms on agar plates typical of those formed by strain NCIB 3610 (Fig. 3D). Therefore, the *B. subtilis epsL* deletion strain was genetically complemented with the PGT coding sequences from *C. jejuni* and *C. concisus* (PglC) (Table S1 to 4). The coding sequences were placed under the control of an isopropyl β-D-1-thiogalactopyranoside (IPTG)-inducible promoter and integrated into the chromosome at the ectopic *amyE* gene in the *epsL* deletion strain. The *B. subtilis epsL* coding region was used as a positive control (Fig. 3D and E; Fig. S3). In each case, in the presence of 25 µM IPTG, the genetic complementation of the *epsL* deletion strain by the *pglC* coding region was noted. The presence of *pglC* provided full recovery of the rugose colony biofilm architecture to the *epsL* deletion strain (Fig. 3D). Additionally, recovery of both the area occupied by the mature colony biofilm (Fig. S3B) and surface hydrophobicity (Fig. 3E; Fig. S3C) was observed to a level that was indistinguishable from the analysis of the NCIB 3610 parental strain. Taken together with the bioinformatic analysis, our biochemical and genetic data support the designation of EpsL as a PGT that installs diNAcBac as the first monosaccharide at the reducing end of the *B. subtilis* EPS.

### Substrate specificity of EpsD, the first GT in the EPS pathway

**Fig 4:**
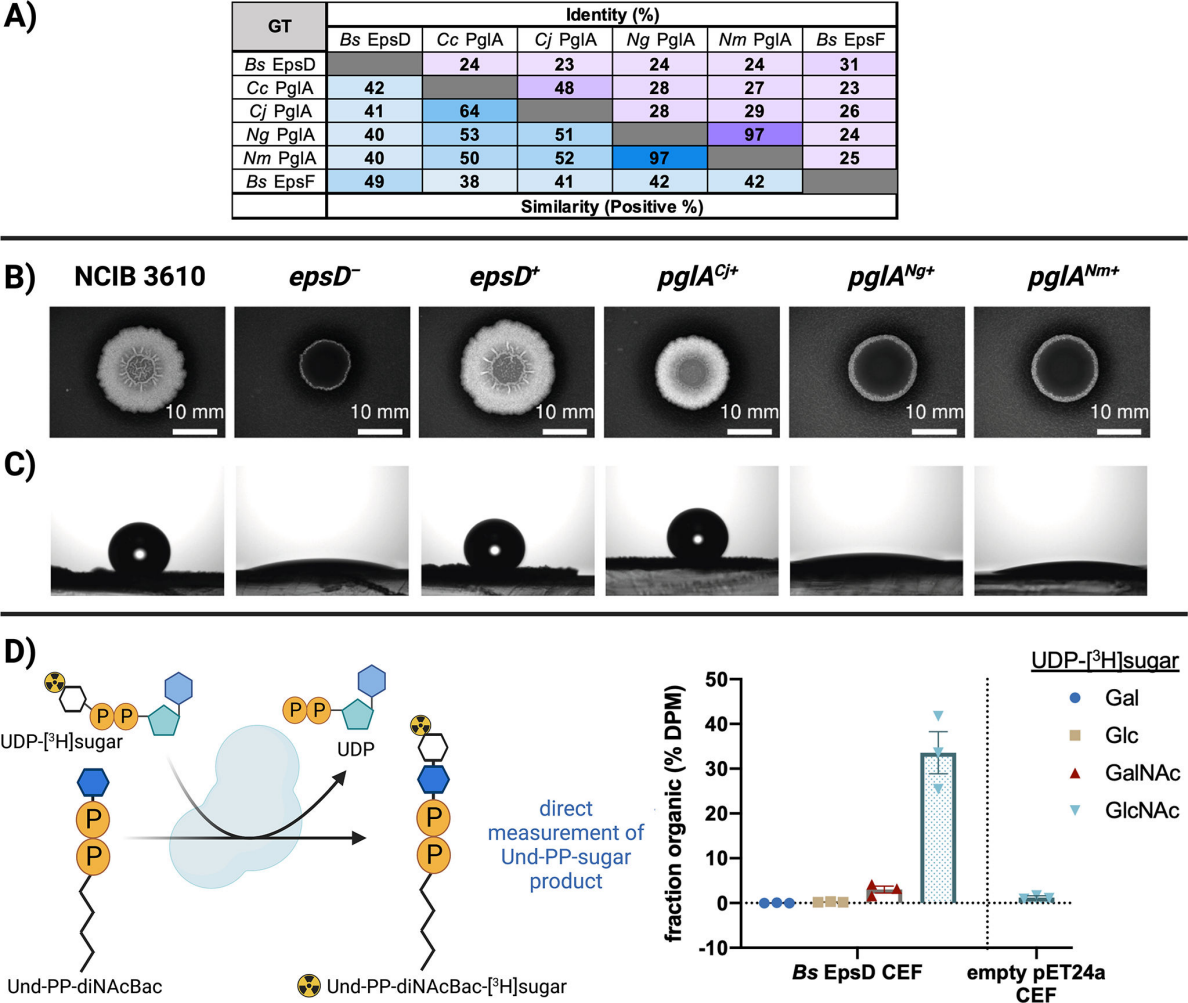
Sequence comparison and biochemical and phenotypic analysis of EpsD. (**A**) Sequence identity of *B. subtilis* EpsD with characterized PglAs from Gram-negative bacteria. Accession numbers: *Bs* EpsD (P71053), *Cc* PglA (A7ZET5), *Cj* PglA (A0A2U0QT38), *Ng* PglA (Q5F602), *Nm* PglA (Q9K1D9), and *Bs* EpsF (P71055). (**B**) and (**C**) Genetic complementation of *Bs ΔepsD* mutant with *pglA* of *Campylobacter* and *Neisseria*. (**B**) represents colony biofilm morphologies of wild-type (*B. subtilis* NCIB 3610), *ΔepsD* mutant (*epsD* ^−^—-NRS5905), and genetically complemented strains (*epsD* ^+^—NRS5930, *pglA^Cj^* ^+^—NRS6605, *pglA^Ng^* ^+^—NRS6619, *pglA^Nm^* ^+^—NRS6620). The colony biofilms were grown at 30°C for 48 h prior to imaging. (**C**) represents the respective sessile water drop analysis of the colony biofilms with a 5 µL water droplet on top. The representative images of wild-type *epsD* ^+^ and *pglA^Cj^* ^+^ were taken after 5 min, whereas the images of *epsD* ^−^ mutant *pglA^Ng^* ^+^ and *pglA^Nm^* ^+^ were taken at 0 min due to extreme hydrophilicity of the surface in the absence of biofilm. (**D**) Biochemical determination of substrate specificity of *Bs* EpsD with Und-PP-diNAcBac as an acceptor substrate in a radioactive-based assay. A negative control of CEF prepared from cells carrying an empty pET24a vector was assayed in parallel. EpsD (*Bs*) activity is background subtracted and reported as the percentage of disintegrations per minute in the organic layer normalized to the total disintegrations per minute per quenched point. Error bars are given for mean ± SEM, *n* = 3.

By determining the first membrane-committed step in the EPS pathway, we were provided with an experimental system where we could use the product of EpsL (Und-PP-diNAcBac) to study the first glycosyl transferase in the pathway. As the structures of glycosyl transferases are relatively similar, it is not possible to predict the substrate specificity from sequence alone. In the *Campylobacter pgl* pathways, the PglA enzyme is responsible for the second step in the glycan biosynthetic pathway, catalyzing the transfer of GalNAc from UDP-GalNAc to Und-PP-diNAcBac (31). There are five GTs encoded by the *epsA-O* operon: EpsD, EpsE, EpsF, EpsH, and EpsJ (Fig. 1A). Of these, EpsD and EpsF are the most similar to PglA at the sequence level (Fig. 4A). EpsD and EpsF are both predicted GT-B-fold enzymes that belong to GT-4 family of retaining GTs in the CAZy classification (33). AlphaFold (34) structural prediction analysis supports that both possess a GT-B-fold, like PglA (Fig. S4). In contrast, the remaining GTs encoded by *epsA-O* operon, EpsE, EpsH, and EpsJ, belong to GT-2 family and are predicted to have GT-A folds. Therefore, based on the sequence similarities of EpsD and EpsF to PglA (Fig. 4A) and their GT structural fold analyses (Fig. S4), we predicted that either EpsD or EpsF could be the first glycosyltransferase in the EPS pathway.

Based on the hypothesis that EpsD or EpsF in *B. subtilis* could carry out the equivalent second step to PglA in *C. jejuni*, we tested whether PglA could functionally substitute for either EpsD or EpsF *in vivo*. We, therefore, investigated the genetic complementation of *B. subtilis epsD* and *epsF* deletion strains by the PglA-coding sequences from *C. jejuni* and other related UDP-Gal transferase enzymes from *Neisseria gonorrhoeae* (*Ng*) and *Neisseria meningitidis* (*Nm*) (Fig. 4B; Fig. S5). The *epsD* and *epsF* deletion strains of *B. subtilis* are unable to form the wild-type rugose, hydrophobic colony biofilms on agar plates (Fig. 4B and C; Fig. S5). The *pglA* genes were placed under the control of an IPTG-inducible promoter and integrated into the chromosome at the ectopic *amyE* gene in the *epsD* and *epsF* deletion strains. The *B. subtilis epsD* and *epsF* coding regions were used as the respective positive controls (see Table S1; Fig. 4B and C; Fig. S5). In the presence of 25 µM IPTG, the genetic complementation of the *epsD* deletion strain by *pglA* gene of *C. jejuni* resulted in partial recovery of biofilm formation, whereas complementation with *pglA* genes from *Neisseria* did not recover the biofilm phenotype (Fig. 4B). In addition to the partial rescue of biofilm phenotype, the complementation of *epsD* deletion strain by *C. jejuni pglA* also recovered the area occupied by the mature colony biofilm (Fig. S5C) and surface hydrophobicity (Fig. 4C; Fig. S5D). The measurements quantified in each case were indistinguishable from those obtained from the analysis of the NCIB 3610 parental strain. In contrast, although the *epsF* deletion strain could be fully complemented by the reintroduction of the *epsF* coding region, expression of the *pglA* genes from *C. jejuni*, *N. gonorrhoeae,* and *N. meningitidis* was unable to recover the biofilm formation (Fig. S5A). This conclusion is supported by AlphaFold modeling of the *Bs* EpsD, EpsF, and *Cc* PglA structures where EpsD and PglA (rsmd 1.4 Å) share an overall higher structural similarity than EpsF and PglA (rsmd 4.1 Å) (Fig. S4B and C).

We next took a biochemical approach to confirm the activity of EpsD by using purified Und-PP-diNAcBac from chemoenzymatic synthesis. To investigate the identity of the UDP-sugar donor for EpsD, we used heterologous expression of EpsD in *E. coli* and isolated CEF (Fig. S6A). Initial attempts to detergent solubilize EpsD were made, and protein was assessed by SDS-PAGE (Fig. S6B). However, the enzyme was no longer active upon solubilization from the CEF (Arbour, Bernstein, Ghosh, Imperiali, unpublished data). Therefore, we investigated the UDP-sugar substrate specificity using the CEF from *E. coli* expressing EpsD using a radioactivity-based assay with Und-PP-diNAcBac, the product of EpsL (Fig. 4D). The panel of sugar donor substrates used for the assay included commercially available UDP-[^3^H]Gal, UDP-[^3^H]Glc, UDP-[^3^H]GalNAc, and UDP-[^3^H]GlcNAc. We determined that in the presence of UDP-[^3^H]GlcNAc, EpsD converts 35% of the UDP-[^3^H]GlcNAc to Und-PP-diNAcBac-[^3^H]GlcNAc. Additionally, under identical conditions, we observed a low, but non-negligible, transfer (4%) of [^3^H]GalNAc to afford Und-PP-diNAcBac-[^3^H]GalNAc (Fig. 4D). No transfer of radioactive sugar was observed with the remaining UDP-sugar substrates or from CEF prepared from *E. coli* carrying the empty pET24a vector (Fig. S6C; Fig. 4D). Moreover, in the presence of UDP-GlcNAc and *Bs* EpsD, we observed a new product by TLC which has a smaller retention factor (*R*_*f*_) than the Und-PP-diNAcBac intermediate (Fig. S2C). Therefore, we conclude that EpsD can use Und-PP-diNAcBac as an acceptor substrate for the transfer of GlcNAc. For structural characterization, the Und-PP-diNAcBac-α1,3-GlcNAc product of *Bs* EpsD was extracted into chloroform and subjected to acid-catalyzed hydrolysis followed by reductive amination with 2-aminobenzamide (2-AB) and sodium cyanoborohydride. This procedure represents a reliable method for glycan analysis and removes complications intrinsic to the size and properties of the undecaprenyl group (31). The 2-aminobenzamide derivative was characterized by fluorescence-based HPLC, negative ion electro-spray ionization (ESI) mass spectrometry, and one-dimensional (1D) and two-dimensional (2D) ^1^H nuclear magnetic resonance (NMR) (Fig. S7) . Regarding the stereochemistry of the new glycosidic linkage, the ^1^H NMR of the 2-AB-labeled disaccharide provides an anomeric spin-spin coupling constant (*J_H_*_1_ *_-H_*_2_) on GlcNAc of 3.93 Hz supporting an α linkage (Fig. S7B) (35). In addition, we examined the sequences of EpsD with PglA from *C. jejuni* and *C. concisus* and the structural overlay of AlphaFold models of EpsD and PglA (*C. concisus*) (Fig. S4A and B). These analyses strongly suggest that EpsD follows a similar mechanistic course to PglA, affording an α-1,3-linkage, which is achieved through a retaining GT mechanism (36). We also note that EpsD displays some substrate promiscuity by accepting UDP-GalNAc as a significantly less preferred substrate (Fig. 4D).

## DISCUSSION

It is extremely challenging to elucidate the structures of complex glycoconjugates directly from bacterial extracts. A case in point is the major polysaccharide found in the extracellular matrix of *B. subtilis* biofilms, which has remained undefined, despite considerable experimentation for many years. This is an important area of research as biofilm formation is a prevalent behavior displayed across multiple microbial species, and EPS production is highly correlated with biofilm formation (37). In this study, we have applied complementary biochemical and genetic approaches to establish the function of essential enzymes that catalyze key early steps in biofilm biosynthesis from the *B. subtilis epsA-O* operon. Overall, the sequences of protein encoded by the operon support the expression of enzymes involved in UDP-sugar biosynthesis as well as several GTs and a PGT with unknown substrate specificity and roles in biofilm biosynthesis (Fig. 1A); however, in the absence of targeted analysis, the EPS pathway cannot be defined.

### EpsL is a functional PGT that utilizes UDP-diNAcBac

Bioinformatic analysis suggested that many of the genes in the *epsA-O* cluster showed similarity to the *pgl* gene cluster, which is responsible for the general protein N-glycosylation pathway in *C. jejuni* (31, 38). As the *pgl* gene cluster had been biochemically characterized and shown to be involved in the biosynthesis of UDP-diNAcBac and a heptasaccharide product containing diNAcBac at the reducing end of the glycan (39), this similarity provided the foundation for exploration of the function of selected enzymes in the *B. subtilis* EPS pathway. Previous sequence analysis and *in vitro* characterization of EpsCNM suggested that these enzymes are responsible for the biosynthesis of UDP-diNAcBac (7 – 9). Sequence analysis also identified EpsL as a close homolog of the *C. jejuni* and *C. concisus* PGTs designated as PglCs, which are now structurally and biochemically well-characterized enzymes (Fig. 2) (22, 23). The identification of a PGT is noteworthy as these enzymes catalyze phosphosugar transfer from UDP-diNAcBac to a polyprenol phosphate carrier as the first membrane-associated step in many glycoconjugate assembly pathways (40).

Thus, we designed a strategy to implement an *in vitro* biochemical activity assay using UndP as the acceptor substrate and a series of [^3^H]-labeled and unlabeled UDP-sugars, including UDP-diNAcBac. Following heterologous expression, solubilization, and purification, EpsL was used to screen enzyme activity *in vitro*. Complementary assays using either radiolabeled sugars or the UMP-Glo assay were applied to confirm that EpsL prefers UDP-diNAcBac as phosphosugar donor and affords the Und-PP-diNAcBac product (Fig. 3B and C). These *in vitro* biochemical assay results were supported by genetic analyses using biofilm formation as the phenotypic readout. This revealed that the *B. subtilis epsL* deletion mutant could be genetically complemented by the *pglC* coding sequence of *C. jejuni* (Fig. 3D). Thus, we conclude that EpsL catalyzes the first step in the EPS biosynthesis pathway to form Und-PP-diNAcBac. Moreover, we show the first experimental evidence of the function of a UDP-diNAcBac utilizing PGT in a Gram-positive bacterium and the presence of diNAcBac as the first sugar at the reducing end of EPS in *B. subtilis*. These findings are significant; diNAcBac was first discovered in *B. licheniformis* (18); however, to date, the diNAcBac sugar has only been described in N- and O-linked glycoproteins, lipopolysaccharide, and the capsular polysaccharide of diverse Gram-negative bacteria (17).

### EpsD is a UDP-GlcNAc-dependent *N*-acetyl glucosamine transferase in *B. subtilis*

The successful characterization of the first step in the EPS pathway provided the Und-PP-diNAcBac substrate for exploring the next enzyme in the EPS biosynthesis. In this case, although the *epsA-O* gene cluster revealed five candidate GTs with predicted GT-A or GT-B fold, the assignment of structure to functional specificity could not be definitively predicted. However, the similarity of *epsA-O* cluster genes with *C. jejuni* N-glycosylation pathway genes helped us to narrow down the candidates to EpsD and EpsF as possible GTs for the subsequent step in the pathway. Our bioinformatic analysis suggested that both EpsD and EpsF share similarity with PglA of *C. jejuni* and selected *Neisseria* spp. (Fig. 4A), and we additionally knew that both EpsF and EpsD were essential for biofilm formation in *B. subtilis* (5). The possibility that EpsF was the next enzyme in the biosynthetic pathway was ruled out by the inability of *pglA* genes of *C. jejuni* and *Neisseria* spp. to rescue the biofilm formation upon expressing in *epsF* deletion mutant of *B. subtilis* (Fig. S5A). It should be noted that we cannot eliminate the possibility that the PglA proteins from *Neisseria* are unstable when produced in *B. subtilis,* and the lack of complementation is due to protein degradation. However, comparable experiments with EpsD provided new insight as genetic complementation with the *C. jejuni pglA* was able to partially rescue the biofilm-negative phenotype in the *epsD* deletion mutant of *B. subtilis* (Fig. 4B). In contrast, the expression of two *pglA variants*, which catalyze the addition of Gal in the second step of the *Neisseria* pgl pathway (41, 42), did not rescue the phenotype in the *epsD* deletion mutant. Although the partial complementation of p*glA* of *C. jejuni* in *epsD* deletion mutant did not confirm the preference of EpsD for GalNAc, it provided the possibility that the preferred sugar substrate could be the related HexNAc sugar, GlcNAc. This hypothesis was supported by the biochemical approach where the cell envelope fraction of *E. coli* expressing EpsD was used to assess the activity using Und-PP-diNAcBac and four different commercially available ^3^H-labeled UDP-sugars as donor substrates. The *in vitro* assay results provided further insight into the EpsD sugar substrate selectivity; EpsD showed a clear preference for UDP-GlcNAc over the other UDP-sugars tested with significant conversion of UDP-[^3^H]GlcNAc to Und-PP-diNAcBac-[^3^H]GlcNAc (Fig. 4D). This supports the function of EpsD in the second step of the EPS pathway. Interestingly, EpsD was also able to transfer [^3^H]GalNAc to Und-PP-diNAcBac, although with far lower efficiency. This donor substrate promiscuity displayed by EpsD not only explains the partial genetic complementation of *epsD* deletion mutant of *B. subtilis* with p*glA* of *C. jejuni* but also provides insight into the step downstream. As previously established, PglA transfers GalNAc onto Und-PP-diNAcBac in *C. jejuni* N-glycans (31, 43). Thus, the partial complementation observed upon expressing *pglA* in the *B. subtilis epsD* deletion mutant suggests that Und-PP-diNAcBac-GalNAc is not a preferred acceptor for the next GT in the *B. subtilis* EPS biosynthetic pathway, resulting in the observed partial biofilm phenotype. It also suggests possible acceptor substrate promiscuity of the next GT in line.

### Summarizing new insights into the *B. subtilis* EPS biosynthetic pathway

**Fig 5:**
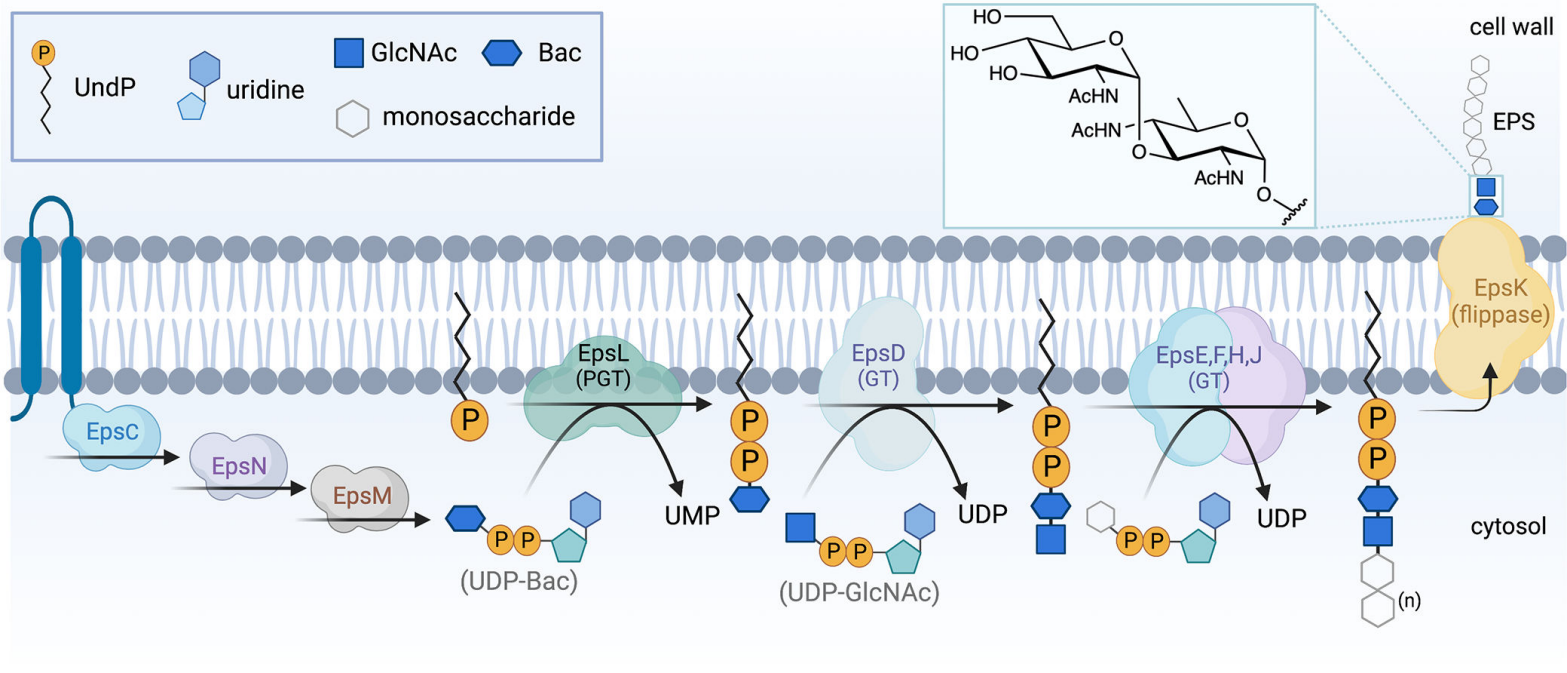
The proposed biofilm matrix exopolysaccharide biosynthetic pathway in *B. subtilis*. EpsCNM synthesizes UDP-diNAcBac, which serves as a donor substrate for EpsL. EpsL transfers diNAcBac onto Und-P, and EpsD catalyzes the second step and transfers GlcNAc from a UDP-GlcNAc sugar donor. The next GTs functioning downstream are to be characterized.

The characterization of EpsL and EpsD in this study has set the foundation for characterizing the remaining GTs in the EPS biosynthesis pathway, which would ultimately enable us to define the EPS sugar composition and structure. Based on the experimental evidence provided in this study, we propose the current EPS glycosylation pathway (Fig. 5). EpsCNM has already been shown to biosynthesize UDP-diNAcBac (7 – 9). EpsL is a PGT that transfers diNAcBac onto Und-P, converting it to Und-PP-diNAcBac. EpsD further extends this glycan by transferring GlcNAc onto the product from EpsL, thus converting it to Und-PP-diNAcBac-GlcNAc. These findings also indicate a divergence in the *B. subtilis* EPS glycosylation pathway after the synthesis of Und-PP-diNAcBac (as diNAcBac-GlcNAc-) compared to *C. jejuni* (diNAcBac-GalNAc-) and *N. gonorrhoeae* (diNAcBac-Gal-) pathways. Homologs of EpsL and EpsD are present broadly across the *B. subtilis* clade. This suggests the presence of similar glycosylation pathways and EPSs in many *Bacillus* species and provides an opportunity to explore the diversity of diNAcBac-containing clusters and the associated EPSs.

### Overarching conclusion

The study of glycoconjugate biosynthesis pathways requires a concerted effort of different approaches as individual bioinformatic, biochemical, and genetic approaches often provide incomplete details. In this study, we establish the sequential characterization of the *B. subtilis* EPS steps by applying biochemical assays and phenotypic screening to the first two membrane-associated processes in the pathway—EpsL and EpsD. The major advantage of addressing steps in the pathway in their biosynthetic order is that the characterization of each enzyme provides the substrate for investigating the following step. Additionally, as enzyme expression and isolation (either in a CEF or in a detergent-solubilized form) are included in the process, it enables the chemoenzymatic synthesis of products for additional analysis and use in related pathways. The established enzyme assays also provide the opportunity for small-molecule inhibitor screening, both individually (EpsL or EpsD) and as biosynthetic partners (EpsL and EpsD). Taken together, these studies set a clear course for analysis of the downstream EPS glycosylation pathway and the development of a complete picture of EPS structure.

## MATERIALS AND METHODS

### Overexpression of *Bs* EpsL and *Bs* EpsD

The EpsD construct was purchased from Twist Bioscience with a C-terminal His_6_ tag in pET24a vector between BamHI and HindIII sites. The EpsL construct was cloned into pET24a vector with a C-terminal His_6_ tag using the Gibson assembly method. Genes that encode for *Bs* EpsL and *Bs* EpsD were codon optimized for *E. coli* expression. The primers used for *Bs* EpsL were 5*'*-GTTTAACTTTAAGAAGGAGATATACATATGATCCTCAAACGGCTGTTCGATCTTACTGCGGCAATC-3*'* (forward) and 5*'*-GTGGTGGTGGTGGTGCGACGAAACGTCACC-3*'* (reverse).

EpsL and EpsD constructs were transformed into BL21(DE3)-RIL cells and overexpressed in the autoinduction medium. A 5 mL seed culture supplemented with 50 µg/mL kanamycin was grown in 50 mM phosphate-aspartate-glucose (MDG) medium (25 mM Na_2_HPO_4_, 25 mM KH_2_PO_4_, 50 mM NH_4_Cl, 5 mM Na_2_SO_4_, 2 mM MgSO_4_, 0.2× trace metals, 0.5% glucose, 0.25% aspartate) (44) at 37°C for 18 h at 225 rpm for each expression. The overnight seed cultures were inoculated into 500 mL autoinduction media [0.1% (wt/vol) tryptone, 0.05% (wt/vol) yeast extract, 2 mM MgSO_4_, 0.05% (vol/vol) glycerol, 0.005% (wt/vol) glucose, 0.02% (wt/vol) α-lactose, 2.5 mM Na_2_HPO_4_, 2.5 mM KH_2_PO_4_, 5 mM NH_4_Cl, 0.5 mM Na_2_SO_4_] in a baffled flask, supplemented with 30 µg/mL chloramphenicol and 90 µg/mL kanamycin. The 500 mL cultures were incubated at 37 °C for 4–5 h at 225 rpm until the bacterial growth reached log phase (OD_600_~0.8–1). The incubation temperature was reduced to 16 °C for the autoinduction of protein expression for 18 h at 225 rpm. The cells were harvested at 3,000 rpm for 25 min at 4 °C. The pellets were washed with 15 mL phosphate buffer saline, flash frozen in LN_2_, and stored at −80°C.

### Preparation of CEF

Cell pellets were resuspended in 50 mL 50 mM HEPES, pH 7.5, 100 mM NaCl, with 25 mg lysozyme (Research Products International, cat #L38100); 25 µL DNAse I (New England BioLabs cat #M0303S); and 50 µL protease inhibitor cocktail (Roche, cat #11836170001). Cells were sonicated twice for 1.5 min (1 s ON/2 s OFF, 50% amplitude), resting on ice for 5 min in between sonication cycles. For EpsL, the lysed cells were centrifuged at 9,000 rpm for 45 min (low-speed spin) using a 45-Ti rotor. The resulting supernatant was transferred to a clean centrifuge tube and centrifuged at 35,000 rpm for 65 min (high-speed spin) in a 45-Ti rotor to pellet the membrane fraction. For EpsD, the lysed cells were directly centrifuged at 35,000 rpm for 65 min (high-speed spin) in a 45-Ti rotor to pellet the membrane fraction. The CEF was homogenized (Dounce) into 12.5 mL 50 mM HEPES, pH 7.5, 100 mM NaCl, with the addition of 14 µL of protease inhibitor cocktail (EMD Millipore, cat #539134).

### Detergent screening of *Bs* EpsL

Small-scale detergent extraction of EpsL was conducted using Anatrace analytical extractor kit (Part# AL-EXTRACT) according to the manufacturer’s protocol with slight modifications. Each detergent at 5× stock solutions was diluted to the working 1× stocks in resuspension buffer (50 mM HEPES, pH 7.5, 100 mM NaCl). The CEF (30 µL of 37 mg/mL *total protein*) of EpsL was diluted with each of the eight detergents (1× stocks, 150 µL) to a final volume of 180 µL. The CEF was solubilized at 4°C for 2 h by a gentle rotation followed by centrifugation at 100,000 × *g* for 1 h using a Beckman-Coulter Ti 42.2 rotor and Beckman-Coulter open-top thick wall polypropylene tubes (7 × 20 mm, Part #343621). The amount of solubilized protein was visualized by SDS-PAGE and Western blotting analysis (Fig. S1A and B).

### Purification of EpsL

The CEF of EpsL in 5.5 mL of 50 mM HEPES, pH 7.5, 100 mM NaCl, was solubilized into 0.09% Triton X-100 (Anapoe-X-100) or 0.03% Octaethylene Glycol Monododecyl Ether (C_12_E_8_) and incubated with rotation at 4 °C for 2 h. The detergent-homogenized sample was centrifuged at 42,000 rpm for 65 min using a 70-Ti rotor. The supernatant was incubated with 1 mL Ni-NTA resin for 1 h at 4 °C. The resin was washed with 10 mL Wash I buffer (50 mM HEPES, pH 7.5, 100 mM NaCl, 15 mM imidazole, 5% glycerol, 0.09% Triton X-100, or 0.03% C_12_E_8_) followed by a wash with 10 mL Wash II buffer (50 mM HEPES, pH 7.5, 100 mM NaCl, 45 mM imidazole, 5% glycerol, 0.09% Triton X-100, or 0.03% C_12_E_8_). EpsL was eluted in 2 × 0.5 mL fractions of elution buffer (50 mM HEPES, pH 7.5, 100 mM NaCl, 500 mM imidazole, 5% glycerol, 0.09% Triton X-100, or 0.03% C_12_E_8_) and immediately desalted using a 5-mL desalting column (Cytiva, HiTrap #17140801) in 50 mM HEPES, pH 7.5, 100 mM NaCl, 5% glycerol, 0.09% TritonX-100, or 0.03% C_12_E_8_. Desalted protein was eluted in 0.5 mL fractions from 1.5 to 3.0 mL. Fractions were pooled, flash frozen in LN_2_, and stored at −80 °C (Fig. S1C).

### General information for UDP-sugars

All commercially available UDP-sugars were purchased from Millipore Sigma. Und-PP-diNAcBac was chemoenzymatically synthesized from UndP and UDP-diNAcBac (see below). The [^3^H]UDP-sugar substrates were diluted from the following specific activities (Ci/mmol) to provide a certain amount of disintegrations per minute (dpm) per assay: UDP-[^3^H]Gal (40 Ci/mmol, 73,000 dpm/assay), UDP-[^3^H]Glc (60 Ci/mmol, 56,000 dpm/assay), UDP-[^3^H]GlcNAc (20 Ci/mmol, 67,000 dpm/assay), UDP-[^3^H]GalNAc (20 Ci/mmol, 65,000 dpm/assay), and UDP-[^3^H]diNAcBac (500 dpm/pmol, 67,000 dpm/assay). Conversion of microcurie (µCi) to dpm is 1 Ci = 2.22 × 10^12^ dpm.

### Western blotting analysis

Protein samples were separated by gel electrophoresis on Biorad 4%–20% gradient gels. The samples were loaded for Western blot and SDS-PAGE analyses. For Western blot analysis, samples were transferred to nitrocellulose at 100 V for 70 min at 4°C. The membrane was then incubated in 25 mL of 3% BSA (0.75 g BSA) in 25 mL Tris-buffered saline with Tween 20 (TBS-T) for 30 min to prevent the nonspecific binding of antibodies to the membrane. For the detection of the His_6_-tagged proteins, the membrane was incubated with a 1:50,000 dilution of mouse anti-His antibody (LifeTein) in TBS-T 3% BSA (5 µL of 1 mg/mL in 25 mL TBS-T with 3% BSA) for 1 h. The membrane was washed with TBS-T for 5 min (5×) followed by incubation with a 1:10,000 dilution of secondary goat antimouse antibody with alkaline phosphatase conjugate in TBS-T buffer (1.5 µL of 0.6 mg/mL in 15 mL TBS-T) for 1 h. The solution was removed, and the membrane was washed with TBS-T (3 × 5 min) followed by TBS (3 × 5 min). The Western blot was developed with alkaline phosphatase substrate (1-step NBT/BCIP) and allowed to develop for 5 min. The blot was washed with water and imaged using a BioRad Molecular Imager Gel Doc XR+ (colorimetric).

### UDP-diNAcBac chemoenzymatic synthesis

We expressed and immobilized the enzymes required for the synthesis of UDP-diNAcBac using previously described methods (45) with slight modifications. The truncated GST-PglF_∆1-130_ (*Cj*) (Addgene ID: 89708) (21) and the PglC-His_6_ (*Ng*) (41) were used to access the UDP-4-ketosugar and the UDP-4-aminosugar, respectively. The 4-aminosugar was synthesized in a single pot, dual-enzyme reaction by immobilizing PglF _∆1-130_ on glutathione resin and PglC (*Ng*) on Ni-NTA resin. *Immobilization of PglF _∆1-130_ on glutathione-resin:* BL21 cells from 0.5 L cultures with overexpressed GST- PglF _∆1-130_ were thawed on ice and resuspended in 40 mL lysis buffer (50 mM HEPES, pH 7.5, 150 mM NaCl, 25 mg lysozyme, 25 µL DNAse I, 40 µL protease inhibitor cocktail) by incubating at 4°C for 30 min with gentle rotation. Cells were sonicated for 90 s with 50% amplitude with 1 s ON and 2 s OFF cycles. The homogenized lysate was transferred into an ultracentrifuge tube and centrifuged at 35,000 rpm in a Ti45 rotor for 1 h at 4°C. The clarified lysate was transferred into a clean tube and incubated with 4 mL glutathione agarose resin (Pierce), pre-equilibrated with working buffer (50 mM HEPES, pH 7.5, 150 mM NaCl). The resin and lysate mixtures were incubated with NAD^+^ at a final concentration of 1 mM for 4 h at 4°C. The resin was transferred to a chromatographic column, and the excess clarified lysate was flowed through the column by gravity. The column was washed with eight column volumes (CV) of working buffer at 4°C to remove excess protein, and the immobilized GST- PglF _∆1-130_ was used immediately. *Immobilization of PglC on Ni-NTA resin:* BL21(DE3) cells from 0.5 L cultures with overexpressed PglC-His_6_ were thawed on ice and resuspended in 40 mL lysis buffer (50 mM HEPES, pH 7.5, 150 mM NaCl, 25 mg lysozyme, 25 µL DNAse I, 40 µL protease inhibitor cocktail) by incubating at 4°C for 30 min with gentle rotation. Cells were sonicated for 90 s with 50% amplitude with 1 s ON and 2 s OFF cycles. The homogenized lysate was transferred into an ultracentrifuge tube and centrifuged at 35,000 rpm in a Ti45 rotor for 1 h at 4°C. The clarified lysate was transferred into a clean tube and incubated with 4 mL Ni-NTA agarose resin (Pierce), pre-equilibrated with working buffer (50 mM HEPES, pH 7.5, 150 mM NaCl). The resin was incubated for 4 h at 4°C with pyridoxal phosphate (PLP) at a final concentration of 1 mM. The resin was transferred to a chromatographic column, and the excess clarified lysate was flowed through the column by gravity. The column was washed with 8 CV of working buffer at 4°C with 15 mM imidazole, followed by 2 CV of working buffer at 4°C without imidazole to remove excess protein. The immobilized PglC-His_6_ was used immediately. *Dual-enzyme synthesis of UDP-4-aminosugar*. Glutathione agarose resin with immobilized GST-PglF_∆1-130_ and Ni-NTA agarose resin with immobilized PglC-His_6_ was resuspended in 1 CV of reaction buffer (50 mM HEPES, pH 8, 150 mM NaCl) and combined in a 50-mL conical tube. UDP-GlcNAc (25 mg) was dissolved in the reaction buffer and added to the resin mix, and NAD^+^ and PLP were added to the resin mix at a final concentration of 0.5 mM each. Additionally, L-glutamic acid was added at a final concentration of 25 mM, and the reaction proceeded for 68–70 h at room temperature with gentle rotation. The resin mix was transferred to a chromatographic column, and the product was collected in the flow-through and combined with the resin washes (2 CV of reaction buffer). Protein present in the flow-through and wash fractions were removed by heating the solution at 60°C for 1 h, followed by centrifugation at 3,200 × *g* for 30 min. The crude UDP-4-aminosugar was purified using a Waters Sep-Pak C18 3 cc Vac Cartridge (Silica-based, 200 mg Sorbent, 55–105 µm, Waters Corp WAT054945). The compound was loaded and eluted in H_2_O (0.1% TFA) and visualized by TLC on glass-backed, silica gel TLC plates (250 µm, F254, SiliCycle TLG-R10014B-323) (UV 254 nM, mobile phase: (5:1:3:1) *n*-BuOH/EtOAc/H_2_O/25% ammonium hydroxide). The combined fractions were lyophilized to provide 22.7 mg of UDP-4-amino sugar (93% yield). The yield was determined by UV-VIS at 262 nm with the extinction coefficient of 10,000 M^−1^ cm^−1^. *Chemical acetylation of UDP-4-aminosugar to access UDP-diNAcBac*. A fraction of the UDP-4-aminosugar stock (2.11 mg) was then dissolved in MeOH (0.5 mM of UDP-sugar in MeOH) followed by the addition of Ac_2_O (40 equivalents) and rotated at ambient temperature for 3 h. The chemical acetylation was monitored by TLC [mobile phase: (5:1:3:1) *n*-BuOH/EtOAc/H_2_O/25% ammonium hydroxide, UV 254 nm] (Fig. S2A), and after complete consumption of starting material, the reaction mixture was concentrated in volume under a stream of N_2_. The resulting crude mixture was purified using a Waters Sep-Pak C18 3 cc Vac Cartridge and eluted in H_2_O (0.1% TFA). The combined fractions containing the desired product were lyophilized to yield UDP-diNAcBac in 72% yield (1.64 mg).

### UDP-[^3^H]diNAcBac enzymatic synthesis and purification

UDP-[^3^H]diNAcBac was synthesized using a modified literature procedure (45, 46) using His_8_-TEV-PglD (*Cj*) (47) to [^3^H]acetylate the UDP-4-aminosugar. To synthesize UDP-[^3^H]diNAcBac, 200 nmol of purified UDP-4-aminosugar was incubated with 2.5 nmol [^3^H]AcCoA (20 Ci/mmol, American Radiolabeled Chemicals) and 26 µM PglD (47) in 50 mM HEPES, pH 7.5, 100 mM NaCl for 30 min at room temperature followed by a chase with an excess of nonradiolabeled AcCoA (247.5 nmol). After overnight rotation, the reaction was supplemented with an additional 13 µM PglD and was allowed to proceed for two more hours. The reaction mixture was prepared for HPLC purification by heating at 60°C for 1 h and centrifugation at 16,000 × *g* for 10 min to precipitate and remove protein. The radiolabeled product was purified on a semi-preparative Dionex CarboPac PA1 HPLC column using the following method: Buffer A = water; Buffer B = 1 M NH_4_HCO_3_; 10%–20% B over 20 min, 20%–70% B over 1 min, 70% B for 10 min, 70%–10% B over 1 min, 10% B for 15 min. The fractions containing UDP-[^3^H]diNAcBac were pooled and lyophilized for several rounds to remove the NH_4_HCO_3_.

### Radioactivity-based biochemical assays on CEF containing overexpressed *Bs* EpsL or detergent-solubilized protein

EpsL (*Bs*) substrate specificity was measured using a radioactively labeled UDP-[^3^H]-sugar panel and an extraction-based assay (48). Enzymatic reactions contained 20 µM UndP (2.5 µL of 200 µM in DMSO), UDP-[^3^H]-sugar (1 µL in H_2_O), and *Bs* EpsL (CEF, 4 mg/mL total protein or 4.48 µM detergent-solubilized EpsL, 2 µL of 12.5× stock) in a final volume of 25 µL of assay buffer (19.5 µL of 50 mM HEPES, pH 7.5, 100 mM NaCl, 0.1% Triton X-100, 5 mM MgCl_2_). The assay contained a final concentration of 10% DMSO. UndP and UDP-[^3^H]-sugar were preincubated in the assay buffer for 30 s, and the reactions were initiated by the addition of EpsL (*Bs*). An aliquot of 15 µL was quenched into 1 mL of 2:1 CHCl_3_/MeOH after 15 min. The organic layer was washed three times with 500 µL PSUP (Pure Solvent Upper Phase = 15 mL CHCl_3_, 240 mL MeOH, 1.83 g KCl, 235 mL H_2_O). The organic layer was mixed with 5 mL Opti-Fluor O scintillation cocktail (PerkinElmer), and the combined aqueous layers were mixed with 5 mL EcoLite Liquid Scintillation Cocktail (MP Biomedicals). All layers were analyzed on a Beckman Coulter LS6500 scintillation counting system with quench compensation.

### UMP-Glo biochemical assays

*B. subtilis* EpsL assays were performed using the Promega UMP-Glo assay, which detects UMP generated over the course of the reaction. The quenching solution was prepared as described by Promega. A UMP-Glo standard curve was obtained using final [UMP] concentrations of 10, 5, 2.5, 1.25 , 0.625, 0.3125, 0.15625, and 0 µM from 10× UMP stocks. The standard curve contained 10% DMSO. The EpsL assays contained 5.6 µM EpsL, 20 µM UndP (10% DMSO final), 0.1% Triton X-100, 50 mM HEPES at pH 7.5, 100 mM NaCl, 5 mM MgCl_2_, and 50 μM UDP-sugar in a final volume of 11 μL. EpsL was preincubated in the reaction mixture lacking the UDP-sugar for 5 min at ambient temperature. Upon the addition of the UDP-sugar, the reaction was allowed to proceed for 30 min before the addition of the quenching solution. The reaction mixture was transferred to a 96-well plate (white, nonbinding surface, Corning). The plate was shaken at low speed for 30 s and incubated for 1 h at 25ºC, and luminescence was read on the plate reader (Fig. S2B). Error bars represent biological triplicate and were calculated with GraphPad Prism 8.

### Bacterial strains and growth conditions

All *B. subtilis* strains used and constructed in this study are listed in Table S1. *E. coli* and *B. subtilis* strains were routinely grown in LB medium (10 g NaCl, 5 g yeast extract, and 10 g tryptone per liter). Complex colony biofilms were grown on biofilm-promoting minimal agar medium (MSgg) [5 mM potassium phosphate and 100 mM MOPs at pH 7.0 supplemented with a metal mix containing 2 mM MgCl_2_, 0.7 mM CaCl_2_, 50 µM MnCl_2_, 50 µM FeCl_3_,1 µM ZnCl_2_, 2 µM thiamine, 0.5% (v/v) glycerol, and 0.5% (w/v) glutamic acid solidified with 1.5% (w/v) Select Agar (Invitrogen)] (10, 49). The biofilms were grown at 30°C for 48 h. Ectopic gene expression was induced with 25 µM IPTG. When appropriate, the antibiotics were used at the following concentrations: ampicillin: 100 µg/mL and spectinomycin:100 µg/mL.

### Strain construction

All strains, plasmids, and primers used in this study are presented in Table S1 to 3. *E*. *coli* strain MC1061 [*F’lacIQ lacZM15 Tn10 (tet*)] was used for the construction and maintenance of all the plasmids. The custom synthesized genes *pglC^Cc^*, *pglC^Cj^*, *pglA^Cj^*, *pglA^Ng^*, and *pglA^Nm^* were codon optimized for optimum expression in *B. subtilis* and were cloned in pUC57 standard plasmid by Genscript using SalI and SphI restriction sites. Table S4 provides more details of the sequences synthesized. The plasmids received from Genscript were used to digest the synthesized gene and cloned into pDR111 plasmid using SalI and SphI restriction sites to generate plasmids pNW2127, pNW1931, pNW1923, pNW1932, and pNW1933, respectively. The *epsL*, *epsF,* and *epsD* coding sequences of *B. subtilis* were also cloned into pDR111 to generate pNW2100, pNW2109, and pNW2103 plasmids. These plasmids were introduced into *B. subtilis* 168 genome using competent cells generated with standard protocols (50). The plasmids integrated into *B. subtilis* chromosome at the non-essential *amyE* gene locus, and the coding region was placed under the control of IPTG-inducible promoter, Phy-spank. SPP1 phage preparation and transduction to introduce DNA into *B. subtilis* strain NCIB 3610 were conducted as described previously (51).

### Colony biofilm morphology assay

*B. subtilis* strains were streaked on LB agar plates and incubated overnight at 37°C. The following day single colonies were grown in 3 mL of LB broth at 37°C with agitation until an OD_600_ ≈ 1.0. All the cultures were normalized to the same density, and 5 µL of the cultures were spotted onto MSgg media plates, without and with 25 µM IPTG. The plates were incubated at 30°C for 48 h before imaging. For all the strains, three independent biological replicates along with their two technical replicates were set up. Biofilm imaging was performed using an MZ16 FA stereomicroscope (Leica) using LAS version 2.7.1. The images were imported into the OMERO server for data management and analysis (52).

### Quantification of biofilm surface area

To quantify the surface area or footprints of biofilms, Fiji/ImageJ software (53, 54) was used with a recently established macro (55, 56) that uses built-in function of ImageJ to detect biofilm regions. The images of colony biofilms were saved as multiseries Leica .LIF files after stereoscopic imaging. The .LIF file was uploaded to macro in Fiji to import the data, and the batch analysis was done on the brightfield images. The outcome was a summary table of detected surface area of biofilms above the background. A minimum of three biological and two technical replicates were performed for each strain.

### Biofilm hydrophobicity assay

The hydrophobicity of biofilms was tested by measuring the contact angle between the surface of the biofilm grown at 30°C for 48 h and a 5 µL water drop of water, as described previously (57). The measurements were taken 5 min after the initial placement of the water droplet on the biofilm surface using a ThetaLite TL100 optical tensiometer (Biolin Scientific). The measurements were taken at 0 min in case of the absence of biofilm. Contact angles were determined with OneAttension software, using the Young-Laplace equation. Contact angles above 90° are indicative of a hydrophobic surface, whereas contact angles below 90° are considered hydrophilic. A minimum of three biological and two technical replicates were performed for each strain.

### Purification of EpsD

The CEF of EpsD in 5.5 mL of 50 mM HEPES pH 7.5, 100 mM NaCl, was solubilized into 0.09% Triton X-100 (Anapoe-X-100) and incubated with rotation at 4 °C for 2 h. The detergent-homogenized sample was centrifuged at 42,000 rpm for 65 min using a 70-Ti rotor. The supernatant was incubated with 1 mL Ni-NTA resin for 1 h at 4 °C. The resin was washed with 10 mL wash I buffer (50 mM HEPES pH 7.5, 100 mM NaCl, 15 mM imidazole, 5% glycerol, 0.09% Triton X-100) followed by a wash with 10 mL wash II buffer (50 mM HEPES pH 7.5, 100 mM NaCl, 45 mM imidazole, 5% glycerol, 0.09% TritonX-100). EpsD was eluted in 2 × 0.5 mL fractions of elution buffer (50 mM HEPES pH 7.5, 100 mM NaCl, 500 mM imidazole, 5% glycerol, 0.09% TritonX-100) and immediately desalted using a 5 mL desalting column in 50 mM HEPES pH 7.5, 100 mM NaCl, 5% glycerol, 0.09% TritonX-100. Desalted protein was eluted in 0.5 mL fractions from 1.5 to 3.0 mL. Fractions were pooled, flash frozen in LN_2_, and stored at −80 °C (Fig. S6B).

### Und-PP-diNAcBac enzymatic synthesis (*Campylobacter concisus* PglC)

The Und-PP-Bac reaction was set up in a 7 mL scintillation vial. The reaction contained a total volume of 400 µL and consisted of 25 µM UndP, 50 µM UDP-diNAcBac, 100 nM *Cc* PglC, 50 mM HEPES pH 7.5, 100 mM NaCl, 0.1% Triton X-100, and 5 mM MgCl_2_. The reaction contained a final concentration of 10% DMSO. The reaction was initiated by the addition of UDP-diNAcBac and allowed to proceed at ambient temperature for 30 min. The reaction was quenched with 2 mL of (2:1) CHCl_3_/MeOH. The organic layer was washed three times with 500 µL PSUP and concentrated under a stream of N_2_. The crude oil was passed through a mini Na_2_SO_4_ pipette column to remove any remaining water, and the eluted mixture was concentrated under N_2_. The oil was then resuspended in a mixture of (7:1) CHCl_3_/MeOH and loaded on a silica column [CV ~0.25 mL, SilicaFlash Irregular Silica Gel P60, 40–63 µM, 60 Å (SiliCycle)]. The crude product was separated using a mobile phase gradient of 4 CV (7:1) CHCl_3_/MeOH, 4 CV (5:1) CHCl_3_/MeOH, and lastly 6 CV of 100% MeOH. Each fraction (~0.25 mL) was analyzed by TLC (solvent: 65:25:4 CHCl_3_/MeOH/H_2_O) and visualized with CAM staining (0.5 g ceric ammonium sulfate, 12 g ammonium molybdate, 15 mL H_2_SO_4_, 235 mL H_2_O) (Fig. S2B). Subsequently, each fraction was quantified by the UDP-Glo biochemical assay (see below).

### UDP-Glo biochemical assays to quantify Und-PP-diNAcBac

Und-PP-diNAcBac concentration determination assays were performed with *Cc* PglA using the Promega UDP-Glo kit from Promega, which detects UDP generated over the course of the reaction. The quenching solution was prepared as described by Promega. A UDP-Glo standard curve was obtained using final [UDP] concentrations of 10, 5, 2.5, 1.25, 0.625, 0.3125, and 0.15625 µM from 10× UDP stocks in H_2_O. The standard curve contained 10% DMSO. The PglA assays contained 100 nM *Cc*PglA, 0.1% Triton X-100, 50 mM HEPES at pH 7.5, 100 mM NaCl, 5 mM MgCl_2_, 25 µM UDP-GalNAc, and Und-PP-diNAcBac in a final volume of 11 µL. An aliquot (5 µL) of each fraction from the Und-PP-diNAcBac purification (see above) was placed in a 1.7 mL Eppendorf and concentrated using the SpeedVac Vacuum Concentrator (10 min). Each concentrated Und-PP-diNAcBac fraction was resuspended in 1.1 µL of DMSO followed by the addition of assay buffer (7.7 µL). Then *Cc* PglA (1.1 µL of 1 µM) was added to the reaction mixture lacking the UDP-sugar for 2 min at ambient temperature. The reactions were initiated by the addition of UDP-GalNAc (1.1 µL of 250 µM in H_2_O) and quenched with 11 µL of the UDP detection reagent after 30 min. The reaction mixture (20 µL) from each sample was transferred to a 96-well plate (white, nonbinding surface, Corning). The plate was shaken at low speed for 30 s and incubated for 1 h at 25°C, and luminescence was read on the plate reader. All luminescence values were background subtracted before converting to UDP.

### Radioactivity-based biochemical assays on *Bs* EpsD CEF

EpsD (*Bs*) substrate specificity was measured using a radioactively-labeled UDP-[^3^H]-sugar panel and an extraction-based assay (48). Enzymatic reactions contained 20 µM Und-PP-Bac (2.5 µL of 200 µM in DMSO), UDP-[^3^H]-sugar (1 µL in H_2_O), and 4 mg/mL EpsD CEF (2 µL of 50 mg/mL *total protein*) in a final volume of 25 µL of assay buffer (19.5 µL of 50 mM HEPES pH 7.5, 100 mM NaCl, 0.1% Triton X-100, 5 mM MgCl_2_). The assay contained a final concentration of 10% DMSO. Und-PP-Bac and UDP-[^3^H]-sugar were preincubated in assay buffer for 30 s, and the reactions were initiated by the addition of EpsD (*Bs*) CEF. An aliquot of 15 µL was quenched into 1 mL of 2:1 CHCl_3_/MeOH after 10 min. The organic layer was washed three times with 500 µL PSUP. The organic layer was mixed with 5 mL Opti-Fluor O scintillation cocktail (PerkinElmer), and the combined aqueous layers were mixed with 5 mL EcoLite Liquid Scintillation Cocktail (MP Biomedicals). All layers were analyzed on a Beckman Coulter LS6500 scintillation counting system with quench compensation.

### Und-PP-diNAcBac-GlcNAc enzymatic synthesis and disaccharide characterization after 2-aminobenzamide (2-AB) labeling

The lipid-linked disaccharide was enzymatically synthesized in dual-enzyme reactions. The reactions were set up in 11 x 7 mL scintillation vials. Each reaction contained a total volume of 1.5 mL and consisted of 265 µM UndP, 300 µM UDP-diNAcBac, 400 µM UDP-GlcNAc, 0.6 µM *Cc* PglC, 1 mg/mL *Bs* EpsD CEF, 50 mM HEPES pH 7.5, 100 mM NaCl, 0.1% Triton X-100, and 5 mM MgCl_2_. The reaction contained a final concentration of 10% DMSO. The reaction was initiated by the addition of the UDP-sugars and allowed to proceed at ambient temperature for 1.5 h. The reaction was quenched with 2 mL of (2:1) CHCl_3_/MeOH. The organic layer was washed with 1 mL PSUP, and the aqueous layer was removed. The aqueous layer was then backextracted with 1 mL (2:1) CHCl_3_/MeOH. The combined organic fractions were washed three times with 1 mL PSUP and concentrated under a stream of N_2_. The 2-AB labeling was performed following a previously established procedure (31) with slight modifications. The Und-PP-diNAcBac-α1,3-GlcNAc product was hydrolyzed with 500 µL of *n*-propanol/2 M trifluoroacetic acid (1:1) and heated at 50°C for 15 min. The resulting solution was evaporated to dryness. The 2-AB labeling reagent was prepared by dissolving 5 mg of 2-AB in 100 µL of acetic acid/DMSO (1:2.3). The entire solution was added to 6 mg of sodium cyanoborohydride to provide the 2-AB labeling reagent. This reagent (17.5 µL) was added to the dried, hydrolyzed disaccharide and heated to 60°C for 2–4 h. The resulting mixture was diluted with H_2_O and purified by fluorescence HPLC. The product was separated from excess dye using a reverse-phase analytical HPLC column (Prozyme GlykoSepR, GKI4727) using solvent A [50 mM ammonium formate (pH 4.4)/10% MeOH (vol/vol)] and solvent B [50 mM ammonium formate (pH 4.4)/20% MeOH (vol/vol)]. Gradient: 0%–100% B over 40 min, flow rate: 0.7 mL/min. The desired product was eluted at 23.2 min (Fig. S7A). The peaks were detected using a fluorescence detector with λ_ex_ = 330 nm and λ_em_ = 420 nm, collected, lyophilized, and analyzed by ESI(-)MS and 1D and 2D NMR (Fig. S7B and C).

## SUPPLEMENTAL MATERIAL

Figure S1 mbio.00948-23-s0001.docx EpsL gels.

Figure S2 mbio.00948-23-s0002.docx TLC analysis.

Figure S3 mbio.00948-23-s0003.docx
*Bs*-EpsL phenotypic analysis.

Figure S4 mbio.00948-23-s0004.docx AlphaFold models.

Figure S5 mbio.00948-23-s0005.docx
*Bs*-EpsD phenotypic analysis.

Figure S6 mbio.00948-23-s0006.docx EpsD gels.

Figure S7 mbio.00948-23-s0007.docx 2AB-HPLC and 1D and 2D ^1^H NMR.

Supplemental Tables mbio.00948-23-s0008.docx Tables S1 to S4.

ASM does not own the copyrights to Supplemental Material that may be linked to, or accessed through, an article. The authors have granted ASM a non-exclusive, world-wide license to publish the Supplemental Material files. Please contact the corresponding author directly for reuse.

## References

[1] Sharma D , Misba L , Khan AU . 2019. Antibiotics versus Biofilm: an emerging battleground in microbial communities. Antimicrob Resist Infect Control 8:76. doi:10.1186/s13756-019-0533-3 31131107 PMC6524306

[2] Vlamakis H , Chai Y , Beauregard P , Losick R , Kolter R . 2013. Sticking together: building a Biofilm the Bacillus subtilis way. Nat Rev Microbiol 11:157–168. doi:10.1038/nrmicro2960 23353768 PMC3936787

[3] Arnaouteli S , Bamford NC , Stanley-Wall NR , Kovács ÁT . 2021. Bacillus subtilis Biofilm formation and social interactions. Nat Rev Microbiol 19:600–614. doi:10.1038/s41579-021-00540-9 33824496

[4] Branda SS , Chu F , Kearns DB , Losick R , Kolter R . 2006. A major protein component of the Bacillus subtilis Biofilm matrix. Mol Microbiol 59:1229–1238. doi:10.1111/j.1365-2958.2005.05020.x 16430696

[5] Roux D , Cywes-Bentley C , Zhang YF , Pons S , Konkol M , Kearns DB , Little DJ , Howell PL , Skurnik D , Pier GB . 2015. Identification of poly-N-acetylglucosamine as a major polysaccharide component of the Bacillus subtilis Biofilm matrix. J Biol Chem 290:19261–19272. doi:10.1074/jbc.M115.648709 26078454 PMC4521046

[6] Irnov I , Winkler WC . 2010. A regulatory RNA required for antitermination of Biofilm and capsular polysaccharide operons in Bacillales. Mol Microbiol 76:559–575. doi:10.1111/j.1365-2958.2010.07131.x 20374491

[7] Kaundinya CR , Savithri HS , Krishnamurthy Rao K , Balaji PV . 2018. In vitro characterization of N-terminal truncated EpsC from Bacillus subtilis 168, a UDP-N-acetylglucosamine 4,6-dehydratase. Arch Biochem Biophys 657:78–88. doi:10.1016/j.abb.2018.09.005 30222950

[8] Kaundinya CR , Savithri HS , Rao KK , Balaji PV . 2018. EpsN from Bacillus subtilis 168 has UDP-2,6-dideoxy 2-acetamido 4-keto glucose aminotransferase activity in vitro. Glycobiology 28:802–812. doi:10.1093/glycob/cwy063 29982582

[9] Kaundinya CR , Savithri HS , Rao KK , Balaji PV . 2018. EpsM from Bacillus subtilis 168 has UDP-2,4,6-trideoxy-2-acetamido-4-amino glucose acetyltransferase activity in vitro. Biochem Biophys Res Commun 505:1057–1062. doi:10.1016/j.bbrc.2018.09.185 30314705

[10] Branda SS , González-Pastor JE , Ben-Yehuda S , Losick R , Kolter R . 2001. Fruiting body formation by Bacillus subtilis. Proc Natl Acad Sci U S A 98:11621–11626. doi:10.1073/pnas.191384198 11572999 PMC58779

[11] Zhu B , Stülke J . 2018. Subtiwiki in 2018: from genes and proteins to functional network annotation of the model organism Bacillus subtilis. Nucleic Acids Res 46:D743–D748. doi:10.1093/nar/gkx908 29788229 PMC5753275

[12] Guttenplan SB , Blair KM , Kearns DB . 2010. The EpsE Flagellar clutch is bifunctional and synergizes with EPS biosynthesis to promote Bacillus subtilis Biofilm formation. PLoS Genet 6:e1001243. doi:10.1371/journal.pgen.1001243 21170308 PMC3000366

[13] Cairns LS , Hobley L , Stanley-Wall NR . 2014. Biofilm formation by Bacillus subtilis: new insights into regulatory strategies and assembly mechanisms. Mol Microbiol 93:587–598. doi:10.1111/mmi.12697 24988880 PMC4238804

[14] Chai Y , Beauregard PB , Vlamakis H , Losick R , Kolter R . 2013. Galactose metabolism plays a crucial role in Biofilm formation by Bacillus Subtilis. mBio 4:e00555-12. doi:10.1128/mBio.00555-12 PMC3419520 22893383

[15] Jones SE , Paynich ML , Kearns DB , Knight KL . 2014. Protection from intestinal inflammation by bacterial exopolysaccharides. J Immunol 192:4813–4820. doi:10.4049/jimmunol.1303369 24740503 PMC4018721

[16] Azulay DN , Abbasi R , Ben Simhon Ktorza I , Remennik S , Reddy M A , Chai L . 2018. Biopolymers from a bacterial extracellular matrix affect the morphology and structure of calcium carbonate crystals. Crystal Growth Des 18:5582–5591. doi:10.1021/acs.cgd.8b00888

[17] Morrison MJ , Imperiali B . 2014. The renaissance of bacillosamine and its derivatives: pathway characterization and implications in pathogenicity. Biochemistry 53:624–638. doi:10.1021/bi401546r 24383882 PMC3951908

[18] Sharon N . 2007. Celebrating the golden anniversary of the discovery of bacillosamine, the diamino sugar of a Bacillus. Glycobiology 17:1150–1155. doi:10.1093/glycob/cwm089 17717023

[19] Schoenhofen IC , McNally DJ , Vinogradov E , Whitfield D , Young NM , Dick S , Wakarchuk WW , Brisson J-R , Logan SM . 2006. Functional characterization of dehydratase/aminotransferase pairs from Helicobacter and Campylobacter: enzymes distinguishing the pseudaminic acid and bacillosamine biosynthetic pathways. J Biol Chem 281:723–732. doi:10.1074/jbc.M511021200 16286454

[20] Olivier NB , Imperiali B . 2008. Crystal structure and catalytic mechanism of PglD from Campylobacter jejuni. J Biol Chem 283:27937–27946. doi:10.1074/jbc.M801207200 18667421 PMC2562079

[21] Olivier NB , Chen MM , Behr JR , Imperiali B . 2006. In vitro biosynthesis of UDP-N,N‘-diacetylbacillosamine by enzymes of the Campylobacter jejuni general protein Glycosylation system. Biochemistry 45:13659–13669. doi:10.1021/bi061456h 17087520 PMC2542654

[22] Ray LC , Das D , Entova S , Lukose V , Lynch AJ , Imperiali B , Allen KN . 2018. Membrane association of monotopic phosphoglycosyl transferase underpins function. Nat Chem Biol 14:538–541. doi:10.1038/s41589-018-0054-z 29769739 PMC6202225

[23] Das D , Kuzmic P , Imperiali B . 2017. Analysis of a dual domain phosphoglycosyl transferase reveals a ping-pong mechanism with a covalent enzyme intermediate. Proc Natl Acad Sci U S A 114:7019–7024. doi:10.1073/pnas.1703397114 28630348 PMC5502628

[24] O’Toole KH , Imperiali B , Allen KN . 2021. Glycoconjugate pathway connections revealed by sequence similarity network analysis of the monotopic phosphoglycosyl transferases. Proc Natl Acad Sci U S A 118:e2018289118. doi:10.1073/pnas.2018289118 33472976 PMC7848588

[25] Entova S , Billod J-M , Swiecicki J-M , Martín-Santamaría S , Imperiali B . 2018. Insights into the key determinants of membrane protein topology enable the identification of new monotopic folds. Elife 7:e40889. doi:10.7554/eLife.40889 30168796 PMC6133551

[26] Rausch M , Deisinger JP , Ulm H , Müller A , Li W , Hardt P , Wang X , Li X , Sylvester M , Engeser M , Vollmer W , Müller CE , Sahl HG , Lee JC , Schneider T . 2019. Coordination of capsule assembly and cell wall biosynthesis in Staphylococcus aureus. Nat Commun 10:1404. doi:10.1038/s41467-019-09356-x 30926919 PMC6441080

[27] Lukose V , Luo L , Kozakov D , Vajda S , Allen KN , Imperiali B . 2015. Conservation and covariance in small bacterial phosphoglycosyltransferases identify the functional catalytic core. Biochemistry 54:7326–7334. doi:10.1021/acs.biochem.5b01086 26600273 PMC5483379

[28] Walvoort MTC , Lukose V , Imperiali B . 2016. A modular approach to phosphoglycosyltransferase inhibitors inspired by nucleoside antibiotics. Chem Eur J 22:3856–3864. doi:10.1002/chem.201503986 26662170 PMC5506376

[29] Das D , Walvoort MTC , Lukose V , Imperiali B . 2016. A rapid and efficient luminescence-based method for assaying phosphoglycosyltransferase enzymes. Sci Rep 6:33412. doi:10.1038/srep33412 27624811 PMC5022061

[30] Dodge GJ , Bernstein HM , Imperiali B . 2023. A Generalizable protocol for expression and purification of membrane-bound bacterial phosphoglycosyl transferases in liponanoparticles. Protein Expr Purif 207:106273. doi:10.1016/j.pep.2023.106273 37068720 PMC10348885

[31] Glover KJ , Weerapana E , Imperiali B . 2005. In vitro assembly of the undecaprenylpyrophosphate-linked heptasaccharide for Prokaryotic N-linked Glycosylation. Proc Natl Acad Sci U S A 102:14255–14259. doi:10.1073/pnas.0507311102 16186480 PMC1242339

[32] Anderson AJ , Dodge GJ , Allen KN , Imperiali B . 2023. Co-conserved sequence motifs are predictive of substrate specificity in a family of Monotopic Phosphoglycosyl Transferases. Protein Sci 32:e4646. doi:10.1002/pro.4646 37096962 PMC10186338

[33] Cantarel BL , Coutinho PM , Rancurel C , Bernard T , Lombard V , Henrissat B . 2009. The carbohydrate-active enzymes database (CAZy): an expert resource for glycogenomics. Nucleic Acids Res 37:D233–D238. doi:10.1093/nar/gkn663 18838391 PMC2686590

[34] Jumper J , Evans R , Pritzel A , Green T , Figurnov M , Ronneberger O , Tunyasuvunakool K , Bates R , Žídek A , Potapenko A , Bridgland A , Meyer C , Kohl SAA , Ballard AJ , Cowie A , Romera-Paredes B , Nikolov S , Jain R , Adler J , Back T , Petersen S , Reiman D , Clancy E , Zielinski M , Steinegger M , Pacholska M , Berghammer T , Bodenstein S , Silver D , Vinyals O , Senior AW , Kavukcuoglu K , Kohli P , Hassabis D . 2021. Highly accurate protein structure prediction with alphafold. Nature 596:583–589. doi:10.1038/s41586-021-03819-2 34265844 PMC8371605

[35] Toukach FV , Ananikov VP . 2013. Recent advances in computational predictions of NMR parameters for the structure elucidation of Carbohydrates: methods and limitations. Chem Soc Rev 42:8376–8415. doi:10.1039/c3cs60073d 23887200

[36] Lairson LL , Henrissat B , Davies GJ , Withers SG . 2008. Glycosyltransferases: structures, functions, and mechanisms. Annu Rev Biochem 77:521–555. doi:10.1146/annurev.biochem.76.061005.092322 18518825

[37] Poulin MB , Kuperman LL . 2021. Regulation of Biofilm exopolysaccharide production by cyclic di-guanosine monophosphate. Front Microbiol 12:730980. doi:10.3389/fmicb.2021.730980 34566936 PMC8461298

[38] Szymanski CM , Yao R , Ewing CP , Trust TJ , Guerry P . 1999. Evidence for a system of general protein glycosylation in Campylobacter jejuni. Mol Microbiol 32:1022–1030. doi:10.1046/j.1365-2958.1999.01415.x 10361304

[39] Young NM , Brisson J-R , Kelly J , Watson DC , Tessier L , Lanthier PH , Jarrell HC , Cadotte N , St Michael F , Aberg E , Szymanski CM . 2002. Structure of the N-linked Glycan present on multiple Glycoproteins in the gram-negative bacterium, Campylobacter jejuni. J Biol Chem 277:42530–42539. doi:10.1074/jbc.M206114200 12186869

[40] O’Toole KH , Bernstein HM , Allen KN , Imperiali B . 2021. The surprising structural and mechanistic dichotomy of membrane-associated phosphoglycosyl transferases. Biochem Soc Trans 49:1189–1203. doi:10.1042/BST20200762 34100892 PMC9206117

[41] Hartley MD , Morrison MJ , Aas FE , Børud B , Koomey M , Imperiali B . 2011. Biochemical characterization of the O-linked glycosylation pathway in Neisseria gonorrhoeae responsible for biosynthesis of protein Glycans containing N,N'-diacetylbacillosamine. Biochemistry 50:4936–4948. doi:10.1021/bi2003372 21542610 PMC3108506

[42] Børud B , Viburiene R , Hartley MD , Paulsen BS , Egge-Jacobsen W , Imperiali B , Koomey M . 2011. Genetic and molecular analyses reveal an evolutionary trajectory for Glycan synthesis in a bacterial protein glycosylation system. Proc Natl Acad Sci U S A 108:9643–9648. doi:10.1073/pnas.1103321108 21606362 PMC3111294

[43] Linton D , Dorrell N , Hitchen PG , Amber S , Karlyshev AV , Morris HR , Dell A , Valvano MA , Aebi M , Wren BW . 2005. Functional analysis of the Campylobacter jejuni N-linked protein glycosylation pathway. Mol Microbiol 55:1695–1703. doi:10.1111/j.1365-2958.2005.04519.x 15752194

[44] Studier FW . 2005. Protein production by auto-induction in high-density shaking cultures. Protein Expr Purif 41:207–234. doi:10.1016/j.pep.2005.01.016 15915565

[45] Zamora CY , Schocker NS , Chang MM , Imperiali B . 2017. Chemoenzymatic synthesis and applications of Prokaryote-specific UDP-sugars. Methods Enzymol 597:145–186. doi:10.1016/bs.mie.2017.06.003 28935101 PMC6710627

[46] Hartley MD , Schneggenburger PE , Imperiali B . 2013. Lipid bilayer nanodisc platform for investigating polyprenol-dependent enzyme interactions and activities. Proc Natl Acad Sci U S A 110:20863–20870. doi:10.1073/pnas.1320852110 24302767 PMC3876266

[47] De Schutter JW , Morrison JP , Morrison MJ , Ciulli A , Imperiali B . 2017. Targeting bacillosamine biosynthesis in bacterial pathogens: development of inhibitors to a bacterial amino-sugar acetyltransferase from Campylobacter jejuni. J Med Chem 60:2099–2118. doi:10.1021/acs.jmedchem.6b01869 28182413 PMC5506375

[48] Lukose V , Walvoort MTC , Imperiali B . 2017. Bacterial phosphoglycosyl transferases: initiators of Glycan biosynthesis at the membrane interface. Glycobiology 27:820–833. doi:10.1093/glycob/cwx064 28810664 PMC5881778

[49] Hobley L , Ostrowski A , Rao FV , Bromley KM , Porter M , Prescott AR , MacPhee CE , van Aalten DMF , Stanley-Wall NR . 2013. BslA is a self-assembling bacterial Hydrophobin that coats the Bacillus subtilis Biofilm. Proc Natl Acad Sci U S A 110:13600–13605. doi:10.1073/pnas.1306390110 23904481 PMC3746881

[50] Smith MCM . 1991. Molecular biological methods for Bacillus. FEBS Lett 287:227–227. doi:10.1016/0014-5793(91)80059-C

[51] Verhamme DT , Kiley TB , Stanley-Wall NR . 2007. DegU co-ordinates multicellular behaviour exhibited by Bacillus subtilis. Mol Microbiol 65:554–568. doi:10.1111/j.1365-2958.2007.05810.x 17590234

[52] Allan C , Burel J-M , Moore J , Blackburn C , Linkert M , Loynton S , Macdonald D , Moore WJ , Neves C , Patterson A , Porter M , Tarkowska A , Loranger B , Avondo J , Lagerstedt I , Lianas L , Leo S , Hands K , Hay RT , Patwardhan A , Best C , Kleywegt GJ , Zanetti G , Swedlow JR . 2012. OMERO: flexible, model-driven data management for experimental biology. Nat Methods 9:245–253. doi:10.1038/nmeth.1896 22373911 PMC3437820

[53] Schindelin J , Arganda-Carreras I , Frise E , Kaynig V , Longair M , Pietzsch T , Preibisch S , Rueden C , Saalfeld S , Schmid B , Tinevez J-Y , White DJ , Hartenstein V , Eliceiri K , Tomancak P , Cardona A . 2012. Fiji: an open-source platform for biological-image analysis. Nat Methods 9:676–682. doi:10.1038/nmeth.2019 22743772 PMC3855844

[54] Schneider CA , Rasband WS , Eliceiri KW . 2012. NIH image to imagej: 25 years of image analysis. Nat Methods 9:671–675. doi:10.1038/nmeth.2089 22930834 PMC5554542

[55] Eigentler L , Ball G . 2021. Code for Eigentler et al. founder cell configuration drives competitive outcome within colony biofilms. Zenodo. doi:10.5281/zenodo.5041981 PMC9122948 35121821

[56] Eigentler L , Kalamara M , Ball G , MacPhee CE , Stanley-Wall NR , Davidson FA . 2022. Founder cell configuration drives competitive outcome within colony Biofilms. ISME J 16:1512–1522. doi:10.1038/s41396-022-01198-8 35121821 PMC9122948

[57] Kalamara M , Abbott JC , MacPhee CE , Stanley-Wall NR . 2021. Biofilm hydrophobicity in environmental isolates of Bacillus subtilis. Microbiology (Reading) 167:001082. doi:10.1099/mic.0.001082 34486975

