# Supplementary material for: Defining early steps in Bacillus subtilis biofilm biosynthesis
Source: mBio. 2023 Aug 31;14(5):e00948-23. doi: 10.1128/mbio.00948-23 (PMC10653937; doi:10.1128/mbio.00948-23)
Supplement: Figure S1 — EpsL gels. [file mbio.00948-23-s0001.docx]

**Figure S1.** *Bs* EpsL detergent solubilization screen ([1](#_ENREF_1)) visualized by **A)** SDS-PAGE and **B)** Western blotting analysis*.* Multiple bands recognized by anti-His antibody above 37 kDa are hypothesized to be high molecular weight protein aggregates. Additionally, because these experiments are from the direct detergent solubilization screen, a range of 0.5-2% detergent is loaded onto the gel. It is possible that this excess detergent is causing streaking at the top of the gel, which is supported by the minimal bands in the buffer only lane. **C)** *Bs* EpsL detergent solubilization with Triton X-100 and C_12_E_8_ visualized by SDS-PAGE (Coomassie). Lanes: 1) CEF; 2) solubilized protein; 3) flow through; 4) 45 mM imidazole wash; 5) 500 mM imidazole elution; 6) desalted pure fraction. **D)** CEF produced from BL21(DE3)RIL cells with 1) empty pET24a vector and 2) overexpressed *Bs* EpsL visualized by SDS-PAGE for comparison.

**Reference**

1. Anatrace. Extractor Kit. <https://www.anatrace.com/Products/Kits/Analytic/ANALYTIC-EXTRACTOR-KIT>.
