# Supplementary material for: Defining early steps in Bacillus subtilis biofilm biosynthesis
Source: mBio. 2023 Aug 31;14(5):e00948-23. doi: 10.1128/mbio.00948-23 (PMC10653937; doi:10.1128/mbio.00948-23)
Supplement: Figure S2 — TLC analysis. [file mbio.00948-23-s0002.docx]

**Figure S2.** Thin-layer chromatography (TLC) characterization of sugar-containing compounds used in this study. **A)** Chemoenzymatic synthesis of UDP-diNAcBac. This figure was curated with BioRender.com. TLC of the chemical acetylation reaction of UDP-4-amino sugar. The solvent system used was (5:1:3:1) *n*-BuOH/EtOAc/H_2_O/25% ammonium hydroxide and visualized by UV (254 nm). Lanes: 1) UDP-4-amino sugar starting material, 2) Co-spot, and 3) reaction after 3 h. **B)** *Bs* EpsL reaction after 30 minutes. The Und-PP-Bac reaction was set up in a 7 mL scintillation vial. The reaction contained a total volume of 200 µL and consisted of 25 µM UndP, 50 µM UDP-diNAcBac, 5.6 µM *Bs* EpsL, 50 mM HEPES pH 7.5, 100 mM NaCl, 0.1% Triton X-100 and 5 mM MgCl_2_. The reactions contained a final concentration of 10% DMSO. TLC with a solvent system of (65:25:4) CHCl_3_/MeOH/H_2_O. Lanes: 1) UndP standard, 2) Und-PP-Bac standard produced from *Cc* PglC, 3) Co-spot, and 4) Und-PP-Bac reaction with *Bs* EpsL. TLC is visualized by CAM staining and imaged on a BioRad Molecular Imager Gel Doc XR+. *Note: The reaction conversion cannot be quantified by TLC due to the differences in staining capabilities between UndP and Und-PP-Bac. Representation of conversion is more accurately visualized by UMP Glo^®^. UMP Glo^®^ activity assay of the EpsL Und-PP-Bac reaction after 30 minutes. Error bars are given for mean ± SEM, n = 2. **C)** TLC of the *Bs* EpsD product with a solvent system of (65:25:4) CHCl_3_/MeOH/H_2_O and visualized by CAM staining and imaged on a BioRad Molecular Imager Gel Doc XR+. Lanes: 1) UndP standard, 2) Co-spot, 3) Und-PP-Bac from *Cc* PglC, and 4) Und-PP-Bac-GlcNAc reaction with *Bs* EpsD.
