# Supplementary material for: Defining early steps in Bacillus subtilis biofilm biosynthesis
Source: mBio. 2023 Aug 31;14(5):e00948-23. doi: 10.1128/mbio.00948-23 (PMC10653937; doi:10.1128/mbio.00948-23)
Supplement: Figure S3 — Bs-EpsL phenotypic analysis. [file mbio.00948-23-s0003.docx]

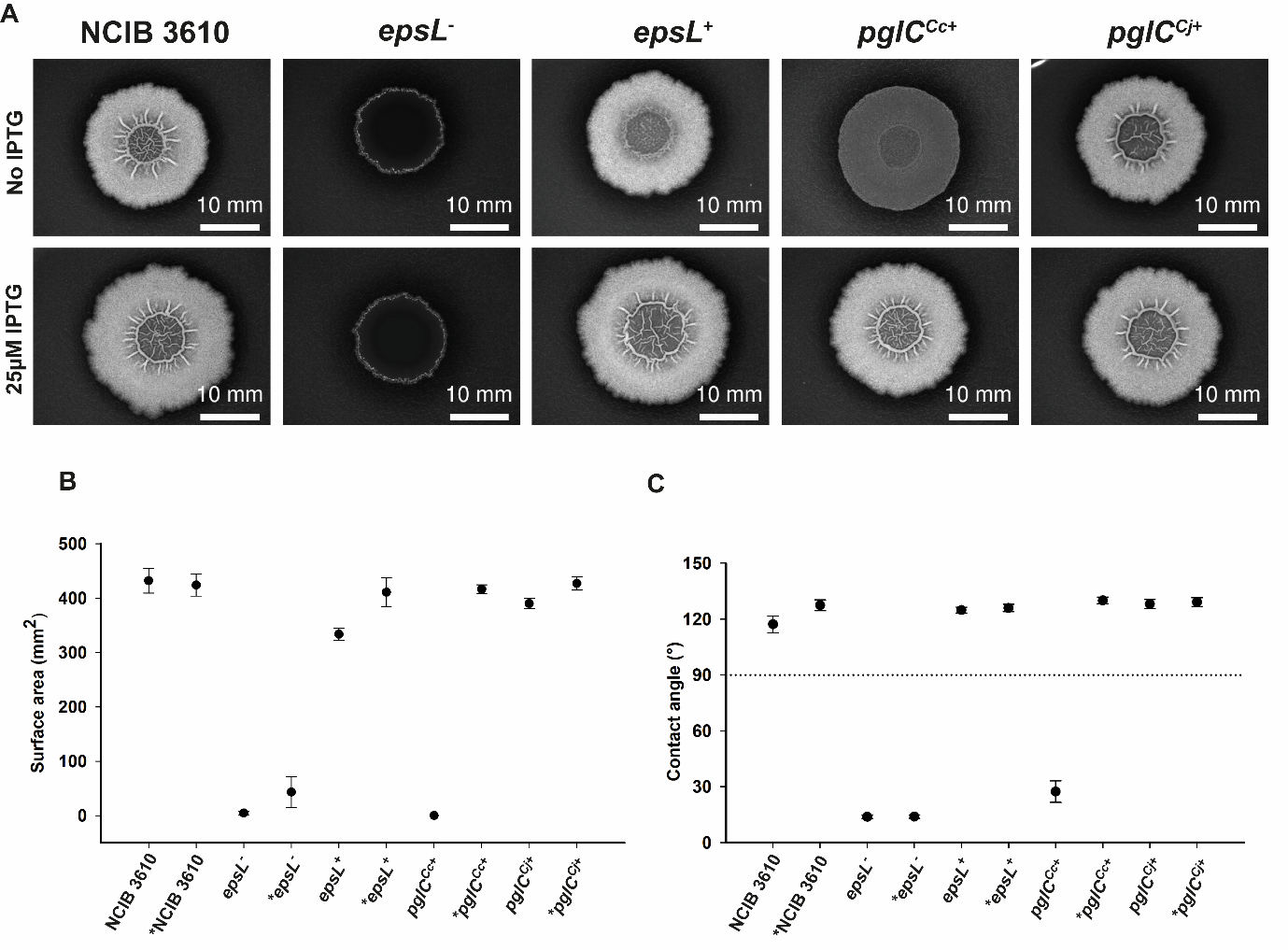


**Figure S3:** Colony biofilm morphology and hydrophobicity upon genetically complementing *ΔepsL-Bs* mutant with *pglC* of *Campylobacter*. (**A**) represents colony biofilm morphologies of wild-type (*B. subtilis* NCIB 3610), *ΔepsL* mutant (*epsL*^-^ - NRS5907) and genetically complemented strains (*epsL*^+^ - NRS5942, *pglC^Cc^*^+^ - NRS6692, *pglC^Cj^*^+^ - NRS6618). The colony biofilms were grown at 30 °C for 48 hours under no IPTG and 25 µM IPTG-induced conditions. (**B**) represents the surface area calculated for the colony biofilm. (**C**) represents the respective sessile water drop analysis of the colony biofilms with a 5 µL water droplet on top. The representative images were taken after 5 minutes, except *epsL*^-^ and *pglC^Cc^*^+^ where the images were taken at 0 minutes due to the extreme hydrophilicity of the surface. Each data point in (**B**) and (**C**) represents the mean value for three biological replicates and their respective two technical replicates. Thus, error bars represent the standard deviation of six replicates. The dotted horizontal reference line in (**C**) represents the 90° contact angle which is a cut-off value for the hydrophobicity of *B. subtilis* biofilm. The data labeled with * on the x-axis represents the values of the biofilm grown under 25 µM IPTG condition.
