# Supplementary material for: Defining early steps in Bacillus subtilis biofilm biosynthesis
Source: mBio. 2023 Aug 31;14(5):e00948-23. doi: 10.1128/mbio.00948-23 (PMC10653937; doi:10.1128/mbio.00948-23)
Supplement: Figure S4 — AlphaFold models. [file mbio.00948-23-s0004.docx]

**

**Figure S4:** Comparison of *B. subtilis* EpsD (P71053) with PglAs from *C. concisus* (A7ZET5) and *C. jejuni* (A0A2U0QT38)*.* **A)** Sequence alignment of EpsD of *B. subtilis* and PglA of *C. concisus* and *C. jejuni.* **B)** Superimposed AlphaFold models of *B. subtilis* EpsD (gold) and *C. concisus* PglA (cyan). Superimposed AlphaFold models of *C. concisus* PglA (cyan) and *B. subtilis* EpsF (magenta, P71055). Highlighted in the inserts are key residues proposed to support intermediates in the retaining GT mechanism by GT-4 family members ([1](#_ENREF_1)).

**Reference**

1. Jumper J, Evans R, Pritzel A, Green T, Figurnov M, Ronneberger O, Tunyasuvunakool K, Bates R, Žídek A, Potapenko A. 2021. Highly accurate protein structure prediction with AlphaFold. Nature 596:583-589.
