# Supplementary material for: Defining early steps in Bacillus subtilis biofilm biosynthesis
Source: mBio. 2023 Aug 31;14(5):e00948-23. doi: 10.1128/mbio.00948-23 (PMC10653937; doi:10.1128/mbio.00948-23)
Supplement: Figure S5 — Bs-EpsD phenotypic analysis. [file mbio.00948-23-s0005.docx]

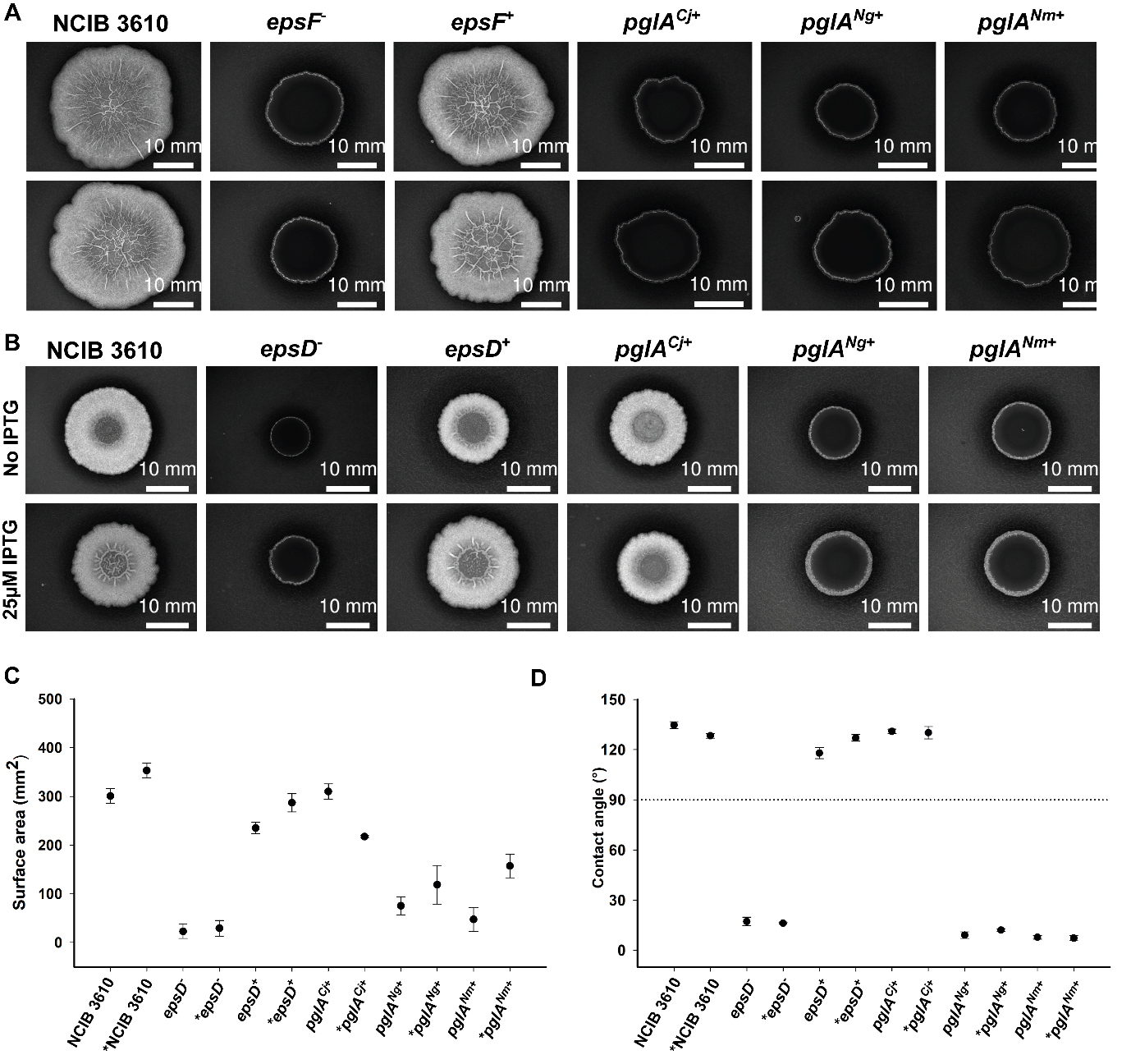


**Figure S5:** Colony biofilm morphology and hydrophobicity upon genetically complementing *ΔepsF-Bs* and *ΔepsD-Bs* mutant with *pglA* of Campylobacter and Neisseria. (**A**) represents colony biofilm morphologies of wild-type (*B. subtilis* NCIB 3610), *ΔepsF* mutant (*epsF*^-^ - NRS5904) and genetically complemented strains (*epsF*^+^ - NRS5961, *pglA^Cj^*^+^ - NRS6628, *pglA^Ng^*^+^ - NRS6629, *pglA^Nm^*^+^ - NRS6630) (**B**) represents colony biofilm morphologies of wild-type (*B. subtilis* NCIB 3610), *ΔepsD* mutant (*epsD*^-^ - NRS5905) and genetically complemented strains (*epsD*^+^ - NRS5930, *pglA^Cj^*^+^ - NRS6605, *pglA^Ng^*^+^ - NRS6619, *pglA^Nm^*^+^ - NRS6620). The colony biofilms were grown at 30 °C for 48 hours under no IPTG and 25 µM IPTG-induced conditions. (**C**) represents the surface area calculated for the colony biofilm. (**D**) represents the respective sessile water drop analysis of the colony biofilms with a 5 µL water droplet on top. The representative images of wild-type, *epsD*^+^ and *pglA^Cj^*^+^ were taken after 5 minutes, whereas the images of *epsD*^-^ mutant, *pglA^Ng^*^+^ and *pglA^Nm^*^+^ were taken at 0 minutes due to extreme hydrophilicity of the surface in absence of biofilm. (**C**) and (**D**) represent the mean value for three biological replicates and their respective two technical replicates. Thus, error bars represent the standard deviation of six replicates. The dotted horizontal reference line in (**D**) represents the 90° contact angle which is a cut-off value for the hydrophobicity of *B. subtilis* biofilm. The data labeled with * on the x-axis represent the values of the biofilm grown under 25 µM IPTG condition.
