# Supplementary material for: Defining early steps in Bacillus subtilis biofilm biosynthesis
Source: mBio. 2023 Aug 31;14(5):e00948-23. doi: 10.1128/mbio.00948-23 (PMC10653937; doi:10.1128/mbio.00948-23)
Supplement: Figure S6 — EpsD gels. [file mbio.00948-23-s0006.docx]

**Figure S6. A)** SDS-PAGE (left panel) and Western blotting analysis (right panel) of *Bs* EpsD cell envelope fraction. **B)** *Bs* EpsD detergent solubilization with Triton X-100 visualized by SDS-PAGE (Coomassie). Lanes: 1) CEF; 2) flow through after solubilization and binding to Ni-NTA resin; 3) 45 mM imidazole wash; 4) 75 mM imidazole wash; 5) 100 mM imidazole wash; 6) 200 mM imidazole wash; and 7) desalted pure fraction from 400 mM imidazole elution. **C)** CEF produced from BL21(DE3)RIL cells with 1) empty pET24a vector and 2) overexpressed *Bs* EpsD visualized by SDS-PAGE to show *E. coli* contaminant bands.
