# Supplementary material for: Defining early steps in Bacillus subtilis biofilm biosynthesis
Source: mBio. 2023 Aug 31;14(5):e00948-23. doi: 10.1128/mbio.00948-23 (PMC10653937; doi:10.1128/mbio.00948-23)
Supplement: Figure S7 — 2AB-HPLC and 1D and 2D 1H NMR. [file mbio.00948-23-s0007.docx]

**Figure S7.** Characterization of 2-aminobenzamide (2-AB)-labeled GlcNAc-Bac disaccharide. **A)** Fluorescence high-performance liquid chromatography (HPLC) and negative-mode ESI mass spectrometry (MS) characterization. **B)** ^1^H Nuclear magnetic resonance (NMR) of the 2-AB-labelled *Bs* EpsD product in D_2_O (600 MHz) with water suppression. **C)** The two-dimensional (2D) NMR (gCOSY) of GlcNAc-Bac-2AB in D_2_O (600 MHz).
