# Supplementary material for: Defining early steps in Bacillus subtilis biofilm biosynthesis
Source: mBio. 2023 Aug 31;14(5):e00948-23. doi: 10.1128/mbio.00948-23 (PMC10653937; doi:10.1128/mbio.00948-23)
Supplement: Supplemental Tables — Tables S1 to S4. [file mbio.00948-23-s0008.docx]

**Table S1. *B. subtilis* strains.**

| **Strain number** | **Genotype^a^** | **Strain Construction/Reference^b^** |
| --- | --- | --- |
| NCIB 3610 | Prototroph | B.G.S.C. |
| 168 | *trpC2* | B.G.S.C. |
| NRS5904 | NCIB 3610 Δ*epsF* | Ref([1](#_ENREF_1)) |
| NRS5905 | NCIB 3610 Δ*epsD* | Ref([1](#_ENREF_1)) |
| NRS5907 | NCIB 3610 Δ*epsL* | Ref([1](#_ENREF_1)) |
| NRS5930 | NCIB 3610 Δ*epsD* *amyE-Phy-spank-epsD-lacI* (spc) | SSP1 NRS5928 → NRS5905 |
| NRS5942 | NCIB 3610 Δ*epsL amyE-Phy-spank*-*epsL*- *lacI* (spc) | SSP1 NRS5941 → NRS5907 |
| NRS5961 | NCIB 3610 Δ*epsF amyE-Phy-spank*-*epsF*- *lacI* (spc) | SSP1 NRS5957→ NRS5904 |
| NRS5992 | NCIB 3610 Δ*epsL amyE-Phy-spank*-*pglC*^Cc^-*lacI* (spc) | SSP1 NRS5990 → NRS5907 |
| NRS6618 | NCIB 3610 Δ*epsL amyE-Phy-spank*-*pglC*^Cj^-*lacI* (spc) | SSP1 NRS6613 → NRS5907 |
| NRS6605 | NCIB 3610 Δ*epsD amyE-Phy-spank*-*pglA*^Cj^-*lacI* (spc) | SSP1 NRS6602 → NRS5905 |
| NRS6619 | NCIB 3610 Δ*epsD amyE-Phy-spank*-*pglA*^Ng^-*lacI* (spc) | SSP1 NRS6614→ NRS5905 |
| NRS6620 | NCIB 3610 Δ*epsD amyE-Phy-spank*-*pglA*^Nm^-*lacI* (spc) | SSP1 NRS6615 → NRS5905 |
| NRS6628 | NCIB 3610 Δ*epsF amyE-Phy-spank*-*pglA*^Cj^-*lacI* (spc) | SSP1 NRS6602 → NRS5904 |
| NRS6629 | NCIB 3610 Δ*epsF amyE-Phy-spank*-*pglA*^Ng^-*lacI* (spc) | SSP1 NRS6614 → NRS5904 |
| NRS6630 | NCIB 3610 Δ*epsF amyE-Phy-spank*-*pglA*^Nm^-*lacI* (spc) | SSP1 NRS6615 → NRS5904 |
| NRS5928 | 168 *trpC2 amyE-Phy-spank-epsD-lacI* (spc) | pNW2100 → 168 |
| NRS5941 | 168 *trpC2 amyE-Phy-spank-epsL-lacI* (spc) | pNW2103 → 168 |
| NRS5957 | 168 *trpC2 amyE-Phy-spank-epsF-lacI* (spc) | pNW2109 → 168 |
| NRS5990 | 168 *trpC2 amyE-Phy-spank*-*pglC*^Cc^-*lacI* (spc) | pNW2127 → 168 |
| NRS6613 | 168 *trpC2 amyE-Phy-spank*-*pglC*^Cj^-*lacI* (spc) | pNW1931 → 168 |
| NRS6602 | 168 *trpC2 amyE-Phy-spank*-*pglA*^Cj^-*lacI* (spc) | pNW1923 → 168 |
| NRS6614 | 168 *trpC2 amyE-Phy-spank*-*pglA*^Ng^-*lacI* (spc) | pNW1932 → 168 |
| NRS6615 | 168 *trpC2 amyE-Phy-spank*-*pglA*^Nm^-*lacI* (spc) | pNW1933 → 168 |

^a^ The relevant genotypes are provided here. Spc, spectinomycin resistance cassette.

^b^ The direction of strain construction is indicated with plasmid DNA or phage (SPP1) (→) recipient using standard techniques. Table S2 provides the details of the plasmids used here. B.S.G.C. represents the Bacillus genetic stock center.

**Table S2: Plasmids used in the genetic section of this study.**

| **Plasmid number** | **Relevant Details** |
| --- | --- |
| pNW2100 | pDR111-*epsD* |
| pNW2103 | pDR111-*epsL* |
| pNW2109 | pDR111-*epsF* |
| pNW2127 | pDR111-*pglC*^Cc (*)^ |
| pNW1931 | pDR111-*pglC*^Cj (*)^ |
| pNW1923 | pDR111-*pglA*^Cj (*)^ |
| pNW1932 | pDR111-*pglA*^Ng (*)^ |
| pNW1933 | pDR111-*pglA*^Nm (*)^ |

^(*)^ Genetic material was synthesized by GenScript. Refer to **Table S4** for full details.

**Table S3: Primers used in the genetic section of this study.**

| **Primer Name** | **Sequence 5’-3’ ^a^** | **Use** |
| --- | --- | --- |
| NSW2592 | GCAGCGTCATTCCAATTTTCAAAAAACAG | *epsD* deletion check |
| NSW2593 | GTGCGGTAGGCACTCGCTCTCA |  |
| NSW2596 | AAATAAAAACCTGCCCGCATCCT | *epsF* deletion check |
| NSW2597 | TTTAGAAAAGCACCGACACTATCAGGTGG |  |
| NSW2608 | GCACCGCTTTTATTTATTCATGCCG | *epsL* deletion check |
| NSW2609 | TTCCGACGTGAGCGCCTTCCT |  |
| NSW872 | AGGTGTGGCATAATGTGTGTAATTGTGAGC | *amyE* pDR111 sequencing |
| NSW873 | TGAACAATCACGAAACAATAATTGGTACGTACG |  |
| NSW2636 | CGGGTCGACTTAGGGGGAGTGAAAATGACGAAAAAGATATTGT | *epsD* cloning |
| NSW2637 | GCGGCATGCTCATACGCTTTTCTCCTTTGTATCCATATCCATGTAC |  |
| NSW2642 | GGCGTCGACACGAAAGGAGCTGTGAATCTTTGATCC | *epsL* cloning |
| NSW2629 | CCGGCATGCTCATGAGGACACATCTCCGCTT |  |
| NSW2653 | CGCGCATGCGAATTCGGAAGGGCTTGTCAAGCATGAATAGC | *epsF* cloning |
| NSW2654 | GGCGCATGCCGGAAAAGGACCATAACCGATGA |  |

**^a^** The underlined sequence represents the restriction enzyme site used for cloning.

**Table S4: Custom synthesized gene sequences.**

| Gene name | Strain Name | Restriction sites added for cloning | Plasmid generated | Original sequence, before codon optimization  Red: Restriction sites  Green: Ribosomal binding site  Black: codon optimized for *B. subtilis* | Codon optimized sequence, received from Genscript  Red: Restriction sites  Green: Ribosomal binding site  Black: codon optimized for *B. subtilis* |
| --- | --- | --- | --- | --- | --- |
| *pglC^Cc^* | *Campylobacter concisus*  GenBank: QPI05301.1 | 5’-SalI- *pglC^Cc^* -SphI-3’ | pNW2127 | GTCGACAAGGAGGTGATCATTAAAAATGTATAGAAATTTTTTAAAGAGAGTGATTGATATTTTGGGAGCTTTGTTTTTGCTCATTTTAACATCGCCTATCATCATAGCAACGGCGATTTTTATCTATTTTAAGGTTAGCCGTGATGTCATTTTTACGCAGGCAAGGCCAGGGCTAAATGAGAAAATTTTTAAAATTTATAAATTTAAGACGATGAGCGACGAGCGTGACGCAAATGGCGAGCTCTTGCCAGATGATCAGCGTCTTGGTAAATTTGGCAAACTTATCCGCTCACTTAGCCTCGATGAGCTGCCACAGCTATTTAACGTGCTAAAGGGCGATATGAGTTTCATCGGACCAAGGCCGCTTTTGGTCGAGTACCTACCCATCTATAACGAAACGCAAAAGCACCGCCACGACGTGCGCCCTGGTATCACGGGTCTAGCGCAGGTAAATGGCAGAAACGCCATAAGCTGGGAGAAAAAATTTGAGTACGACGTCTATTATGCTAAAAATTTAAGCTTTATGCTTGATGTAAAGATCGCTTTGCAGACCATCGAAAAAGTGCTAAAACGAAGTGGTGTCAGCAAAGAGGGGCAGGCGACGACGGAGAAATTTAATGGCAAAAACTAAGCATGC | GTCGACAAGGAGGTGATCATTAAAAATGTACCGTAACTTCCTTAAACGTGTTATCGATATCCTTGGCGCTCTTTTCCTTCTTATCCTTACATCTCCTATCATCATCGCTACAGCTATCTTCATCTACTTCAAAGTTTCTCGTGATGTTATCTTCACACAAGCTCGTCCTGGCCTTAACGAAAAAATCTTCAAAATCTACAAATTCAAAACAATGTCTGATGAACGTGATGCTAACGGCGAACTTCTTCCTGATGATCAACGTCTTGGCAAATTCGGCAAACTTATCCGTTCTCTTTCTCTTGATGAACTTCCTCAACTTTTCAACGTTCTTAAAGGCGATATGTCTTTCATCGGCCCTCGTCCTCTTCTTGTTGAATACCTTCCTATCTACAACGAAACACAAAAACATCGTCATGATGTTCGTCCTGGCATCACAGGCCTTGCTCAAGTTAACGGCCGTAACGCTATCTCTTGGGAAAAAAAATTCGAATACGATGTTTACTACGCTAAAAACCTTTCTTTCATGCTTGATGTTAAAATCGCTCTTCAAACAATCGAAAAAGTTCTTAAACGTTCTGGCGTTTCTAAAGAAGGCCAAGCTACAACAGAAAAATTCAACGGCAAAAACTAAGCATGC |
| *pglC^C^*^j^ | *Campylobacter jejuni* subsp. jejuni 81-176  GenBank: AAD51385.1 | 5’-SalI- *pglC^C^*^j^ -SphI-3’ | pNW1931 | GTCGACAAGGAGGTGATCATTAAAAATGTATGAAAAAGTTTTTAAAAGAATTTTTGATTTTATTTTAGCTTTAGTGCTTTTAGTACTTTTTTCTCCGGTGATTTTAATCACTGCTTTACTTTTAAAAATCACTCAAGGAAGTGTGATTTTCACTCAAAATCGCCCTGGGTTAGATGAAAAAATTTTTAAAATTTATAAATTTAAAACCATGAGCGATGAAAGAGATGAGAAGGGTGAGTTATTAAGCGATGAATTGCGTTTGAAAGCCTTTGGAAAAATTGTTAGAAGCTTAAGTTTGGATGAGCTTTTGCAACTTTTTAATGTTTTAAAAGGGGATATGAGTTTTGTGGGGCCTAGGCCTCTTTTGGTTGAGTATTTATCCCTTTATAATGAAGAGCAAAAATTGCGCCATAAGGTGCGTCCAGGTATAACAGGATGGGCGCAGGTAAATGGCAGAAATGCTATTTCTTGGCAGAAAAAATTCGAACTTGATGTGTATTATGTGAAAAATATTTCTTTTTTGCTTGATTTAAAAATCATGTTTTTAACAGCTTTAAAGGTTTTAAAACGAAGCGGGGTAAGCAAAGAAGGCCATGTTACAACAGAGAAATTTAATGGCAAGAACTGAGCATGC | GTCGACAAGGAGGTGATCATTAAAAATGTATGAAAAAGTTTTTAAAAGAATCTTTGATTTTATCCTTGCTTTAGTTCTGCTTGTGTTATTTTCTCCGGTGATTCTGATCACAGCCTTACTGCTTAAAATTACACAAGGCTCAGTCATCTTTACACAGAATAGACCGGGACTGGATGAAAAAATCTTTAAAATCTACAAATTTAAAACAATGAGCGATGAACGCGATGAAAAAGGCGAATTACTGTCTGATGAACTGAGACTTAAAGCATTTGGAAAAATTGTCCGCTCATTAAGCCTGGATGAACTTCTGCAACTGTTTAACGTTCTTAAAGGCGATATGTCATTTGTGGGACCGAGACCGCTGCTTGTCGAATACCTTAGCCTGTACAACGAAGAACAGAAACTTAGACATAAAGTTCGCCCGGGCATTACAGGATGGGCACAAGTGAATGGCCGCAACGCGATCAGCTGGCAGAAAAAATTTGAACTTGATGTCTACTACGTTAAAAACATTTCATTTCTGCTGGATCTGAAAATCATGTTTCTGACAGCTCTTAAAGTGTTAAAACGCTCTGGCGTCTCAAAAGAAGGACATGTTACAACAGAAAAATTTAATGGAAAAAACTAAGCATGC |
| *pglA^Cj^* | *Campylobacter jejuni* subsp. jejuni 81-176  GenBank: AAD51384.1 | 5’-SalI- *pglA^Cj^* -SphI-3’ | pNW1923 | GTCGACAAGGAGGTGATCATTAAAAATGAGAATAGGATTTTTATCACATGCAGGAGCAAGTATTTATCATTTTAGAATGCCTATTATAAAAGCATTAAAAGATAGAAAAGATGAAGTTTTTGTTATAGTGCCACAAGATGAATACACGCAAAAACTTAGAGATCTTGGCTTAAAAGTAATTGTTTATGAGCTTTCAAGAGCTAGTTTAAATCCTTTTGTGGTTTTAAAGAATTTTTTTTATCTTGCTAAGGTTTTGAAAAATTTAAATCTTGATCTTATTCAAAGTGCGGCACACAAAAGCAATACCTTTGGAATTTTAGCAGCAAAATGGGCAAAAATTCCTTATCGTTTTGCCTTAGTAGAAGGCTTGGGATCTTTTTATATAGATCAAGGTTTTAAGGCAAATTTAGTGCGTTTTGTTATTAATAATCTTTATAAATTAGGTTTTAAATTTGCACACCAATTTATTTTTGTCAATGAAAGTAATGCTGAGTTTATGCGGAATTTAGGATTTAAGGAAAGTAAAATTTGCGTGATAAAATCTGTAGGGATCAATTTAAAAAAATTTTTTCCTATTTATGTAGAATCGGAAAAAAAAGAGCTTTTTTGGAAAAAATTAAACATAGATAAAAAGCCCATTGTGCTTATGATAGCAAGAGCTTTATGGCATAAAGGTGTAAAAGAATTTTATGAAAGTGCTACTATGCTAAAAGACAAAGCAAATTTTGTTTTAGTTGGTGGAAGAGATGAAAATCCTTCTTGTGCGAGTTTGGAGTTTTTAAACTCGGGTGTGGTGCATTATTTGGGTGCTAGAAGTGATATAGTCGAGCTTTTGCAAAATTGTGATATTTTTGTTTTACCAAGCTATAAAGAAGGCTTTCCTGTAAGTGTTTTGGAGGCAAAAGCTTGTGGCAAGGCTATAGTGGTGAGTGATTGTGAAGGTTGTGTAGAGGCTATTTCTAATGCTTATGATGGACTTTGGGCAAAAACAAAAAATGCTAAGGATTTAAGCGAAAAAATTTCACTTTTATTAGAAGATGAAAAATTAAGATTAAATTTAGCTAAAAATGCTGCCCAAGATGCTTTACAATACGATGAAAATAATATCGCACAGCGTTATTTAAAACTTTATGATAGGGTAATTAAGAATGTATGAGCATGC | GTCGACAAGGAGGTGATCATTAAAAATGAGAATTGGCTTTCTTTCACATGCTGGAGCCAGCATCTACCATTTTAGAATGCCGATCATCAAAGCACTGAAAGATCGCAAAGATGAAGTTTTTGTGATTGTCCCGCAAGATGAATATACACAGAAACTGAGAGATTTAGGCCTGAAAGTTATCGTGTATGAATTATCACGCGCAAGCCTGAATCCGTTTGTTGTGCTTAAAAATTTCTTTTATCTTGCGAAAGTGCTGAAAAACCTTAACCTGGATCTGATCCAATCTGCAGCGCATAAATCAAACACATTTGGAATCCTGGCTGCCAAATGGGCAAAAATCCCGTATAGATTTGCGCTGGTCGAAGGCCTTGGATCTTTTTACATCGATCAAGGCTTTAAAGCGAATCTGGTTCGCTTTGTGATCAACAACCTGTACAAACTTGGATTTAAATTTGCTCATCAGTTTATCTTTGTT  AACGAATCAAACGCCGAATTTATGAGAAACCTGGGCTTTAAAGAAAGCAAAATCTGCGTCATCAAATCAGTTGGAATCAACCTGAAAAAATTTTTCCCGATCTATGTGGAAAGCGAAAAGAAAGAACTGTTTTGGAAAAAACTGAACATCGATAAAAAACCGATTGTCCTGATGATCGCACGCGCGCTTTGGCATAAAGGCGTTAAAGAATTTTACGAAAGCGCTACAATGCTGAAAGATAAAGCCAACTTTGTCCTGGTTGGCGGAAGAGATGAAAACCCGTCTTGTGCTTCATTAGAATTTCTGAATTCTGGCGTCGTTCATTATCTTGGAGCCCGCTCAGATATTGTTGAACTGCTTCAAAATTGCGATATCTTTGTGCTGCCGTCATATAAAGAAGGCTTTCCTGTGAGCGTCCTTGAAGCTAAAGCCTGTGGCAAAGCGATTGTGGTCAGCGATTGCGAAGGATGTGTTGAAGCAATCTCTAACGCGTATGATGGACTTTGGGCTAAAACAAAAAACGCCAAAGATCTGAGCGAAAAAATCTCTCTGCTGCTTGAAGATGAAAAACTTCGCTTAAATCTGGCAAAAAACGCAGCGCAAGATGCGCTGCAGTACGATGAAAACAACATCGCTCAGAGATACCTTAAACTGTACGATCGCGTGATCAAAAATGTCTAAGCATGC |
| *pglA^Ng^* | *Neisseria gonorrhoeae* FA1090  GenBank: AAM15778.1 | 5’-SalI- *pglA^Ng^* -SphI-3’ | pNW1932 | GTCGACAAGGAGGTGATCATTAAAAATGAAAATCGTTTTTATCACAACAGTCGCATCCAGCATTTACGGTTTCCGCGCCCCCGTCATTAAAAAATTAATCGGCAAAAACCATCAGGTGTATGCCTTTGTATCGGAGTTTTCCGATAATGAGTTGGACATTATCAGGGAAATGGGGGTTACACCCGTTACCTACCGGTCAAACCGCAGCGGGGTAAACCCGTTTTCCGATATAAAATCCACCTTCCTCATATTTAAAGCACTCAAAAAAATATCGCCGGATTTGGTTTTCCCTTATTTCGCAAAACCCGTGATTTTCGGCACTTTTGCCGCAAAATTGGCAGGCGTGCCCAGAATCGTCGGGATGCTGGAAGGTTTGGGATTCGCATTTACCCCGCAGCCGGAAGGCATACCGTTAAAAACAAAAATAATAAAGGGCATTTTGATTGCCCTGTACCGCATTGCCCTGCCGATGTTGGAAAGCCTGATCGTATTAAACCCCGACGACAAAGACGAGCTGCTGCATCAATACGGCATCAAAATAAAAAACATTCATATTTTGGGCGGAATCGGTCTGGATTTGCGGCAATATCCTTATTCCGAGGCGGATATTCCCGATGAAAAAGAACCCGTAAAATTTCTCTTTATCGGCAGATTTCTGAAAGAAAAGGGGATTGATGATTTTATTCGGGCGGCGGAACAGGTTAAGGGCAAATACCCCGATACGGTTTTTACCGCTTTGGGCGCAATCGACAAATCACGCGGGGGGGGGGGAGATTTAGAACGCTTTATCGCCCGCGATATTATCCGTTTCCCCGGTTTTGTGAACAATGTTTCCGAAGTGATAAAGGCGCATCATATATTCGTATTGCCGTCTTATTATAGGGAAGGCGTTCCCCGAAGCACCCAGGAGGCAATGGCCGTCGGCAGGGCGGTGATTACGACGGATGTCCCCGGATGCAGGGAAACGGTTGCCGACAAGGTCAACGGCTTCCTGATCGAACCTTGGAATCCCCGCATCTTGGCCGAAAAAATGATTTATTTTATCGAAAACAGGGCTGCCGTCCGCCTGATGGCGAATGCAAGTTATGCGATTGCCAAAGATAAATTCGATGCCGAAAAAGTCGATTTGAAATTTCTCGATATTTTGAAGGCGTAAGCATGC | GTCGACAAGGAGGTGATCATTAAAAATGAAAATTGTGTTTATCACAACAGTCGCATCAAGCATTTATGGCTTTCGCGCGCCGGTCATCAAAAAACTTATCGGAAAAAACCATCAAGTCTACGCATTTGTTTCTGAATTTTCAGATAACGAACTGGATATCATCAGAGAAATGGGCGTTACACCGGTGACATATAGAAGCAATCGCTCTGGAGTTAACCCGTTTTCAGATATCAAAAGCACATTTCTGATCTTTAAAGCTCTGAAGAAAATTTCTCCGGATTTAGTTTTTCCGTATTTTGCTAAACCGGTGATCTTTGGCACATTTGCAGCGAAACTGGCCGGCGTGCCGCGCATTGTCGGAATGCTTGAAGGCTTAGGATTTGCCTTTACACCGCAGCCGGAAGGCATTCCGCTGAAAACAAAAATCATCAAAGGAATCCTGATCGCTCTTTATAGAATTGCCTTACCGATGCTGGAATCACTTATCGTGTTAAATCCGGATGATAAAGATGAACTGCTTCATCAATACGGCATCAAAATCAAAAACATCCATATCCTTGGCGGAATTGGATTAGATCTGCGCCAGTATCCGTATAGCGAAGCGGATATTCCGGATGAAAAAGAA  CCGGTCAAATTTCTGTTTATCGGCCGCTTTCTGAAAGAAAAAGGAATCGATGATTTTATCAGAGCTGCCGAACAAGTCAAAGGCAAATATCCGGATACAGTTTTTACAGCACTGGGAGCGATCGATAAAAGCAGAGGCGGAGGCGGAGATCTTGAAAGATTTATTGCACGCGATATTATCAGATTTCCGGGCTTTGTCAACAACGTTTCTGAAGTGATCAAAGCGCATCATATCTTTGTGTTACCGTCTTATTATCGCGAAGGAGTCCCGAGATCAACACAGGAAGCTATGGCCGTTGGCCGCGCTGTGATTACAACAGATGTTCCGGGATGCAGAGAAACAGTCGCCGATAAAGTTAACGGATTTCTTATCGAACCGTGGAACCCGAGAATCCTGGCTGAAAAAATGATCTACTTTATCGAAAATCGCGCAGCGGTTAGACTGATGGCAAACGCGAGCTATGCTATCGCCAAAGATAAATTTGATGCAGAAAAAGTGGATCTGAAATTTCTTGATATTTTAAAAGCGTAAGCATGC |
| *pglA^Nm^* | *Neisseria meningitidis* C311#3  GenBank: QXZ29626.1 | 5’-SalI- *pglA^Nm^* -SphI-3’ | pNW1933 | GTCGACAAGGAGGTGATCATTAAAAATGAAAATCGTTTTTATCACAACAGTCGCATCCAGCATTTACGGTTTCCGCGCCCCCGTCATTAAAAAATTAATCGGCAAAAACCATCAGGTGTATGCCTTTGTATCGGAGTTTTCCGACAATGAATTGGATATTATCAGGGAAATGGGGGTTACACCCGTTACCTACCGTTCAAACCGCAGCGGGCTGAACCCGTTTTCGGATATAAAATCCACCTTCCTCATCTTTAAAGAACTCAAAAAAATATCGCCGGATTTGGTTTTCCCTTATTTCGCAAAACCCGTGATTTTCGGCACTTTTGCCGCAAAACTGGCAGGCGTGCCCAGAATCGTCGGGATGCTGGAAGGTTTGGGATTCGCATTTACCCCGCAGCCGGAAGGCATACCGTTAAAAACAAAAATCATAAAGGGGATTTTGATTGCCTTATACCGCATTGCCCTGCCGATGTTGGAAAGCCTGATTGTATTAAACCCCGACGACAAAGACGAACTGACGGACAAATACGGCATCAAAATAAAAAACATCCATATTTTGGGCGGAATCGGTCTGGATTTGCGGCAATATCCTTATTCCGAGGCGGATATTCCCGATGAAAAAGAACCCGTAAAATTCCTCTTTATCGGCAGATTTCTGAAAGAAAAGGGGATTGATGATTTTATTCGGGCGGCGGAACAGGTTAAGGACAAATACCCCGATACGGTTTTTACCGCTTTGGGCGCAATCGACAAATCACGCGGGGGGGGGGGCGATTTGGAACGGCTTGCCGCCCGCGATATTATCCGTTTCCCCGGTTTTGTGAACAATGTTTCCGAAGTGATAAAAGAACATCATATATTCGTATTGCCGTCTTATTATAGGGAAGGCGTTCCCCGAAGCACTCAGGAGGCAATGGCCGTCGGCAGGGCAGTGATTACGACGGATGTCCCCGGATGCAGGGAAACGGTCGCCGACAAGGTCAACGGCTTCCTGATCGAGCCTTGGAATCCCCGCATCTTGGCCGAAAAAATGATTTATTTTATCGAAAACAGGGAAGCCGTCCGCCTGATGGGGAATGCAAGTTATGCGATTGCCAAAGATAAATTCGATGCCGAAAAAGTCGATTTGAAATTGCTCGATATTTTGAAGGCGTAAGCATGC | GTCGACAAGGAGGTGATCATTAAAAATGAAAATTGTGTTTATCACAACAGTCGCATCAAGCATTTATGGCTTTCGCGCGCCGGTGATCAAAAAACTGATCGGAAAAAACCATCAAGTCTACGCTTTTGTTTCTGAATTTTCAGATAACGAACTGGATATCATCAGAGAAATGGGCGTTACACCGGTGACATATAGAAGCAATCGCTCTGGACTGAACCCGTTTTCAGATATCAAAAGCACATTTTTAATCTTTAAAGAACTGAAGAAAATTTCTCCGGATCTGGTTTTTCCGTATTTTGCTAAACCGGTGATCTTTGGCACATTTGCAGCGAAACTTGCCGGCGTGCCGCGCATTGTCGGAATGCTTGAAGGCTTAGGATTTGCATTTACACCGCAGCCGGAAGGCATCCCGCTTAAAACAAAAATCATCAAAGGAATCCTGATCGCACTTTATAGAATTGCGTTACCGATGCTGGAATCACTTATCGTCTTAAATCCGGATGATAAAGATGAACTTACAGATAAATACGGCATCAAAATCAAAAACATTCATATCTTAGGCGGAATTGGATTAGATCTGCGCCAATATCCGTATAGCGAAGCGGATATTCCGGATGAAAAAGAA  CCGGTTAAATTTCTTTTTATCGGCCGCTTTTTAAAAGAAAAAGGAATCGATGATTTTATCAGAGCTGCCGAACAGGTCAAAGATAAATACCCGGATACAGTTTTTACAGCTCTTGGAGCCATTGATAAAAGCAGAGGCGGAGGCGGAGATCTGGAAAGACTTGCAGCGCGCGATATTATCAGATTTCCGGGCTTTGTCAACAACGTTTCTGAAGTGATCAAAGAACATCATATCTTTGTGTTACCGTCTTATTATCGCGAAGGAGTCCCGAGATCAACACAAGAAGCTATGGCCGTTGGCCGCGCTGTGATTACAACAGATGTTCCGGGATGCAGAGAAACAGTCGCCGATAAAGTTAACGGCTTTTTAATCGAACCGTGGAACCCGAGAATCCTGGCAGAAAAAATGATCTACTTTATCGAAAACCGCGAAGCTGTTAGATTAATGGGA  AACGCCAGCTATGCAATCGCGAAAGATAAATTTGATGCAGAAAAAGTGGATCTGAAACTGCTTGATATTCTTAAAGCGTAAGCATGC |

**Reference**

1. Roux D, Cywes-Bentley C, Zhang YF, Pons S, Konkol M, Kearns DB, Little DJ, Howell PL, Skurnik D, Pier GB. 2015. Identification of Poly-N-Acetylglucosamine as a Major Polysaccharide Component of the *Bacillus subtilis* Biofilm Matrix. J Biol Chem 290:19261-72.
